# Convergent evolution of ST2 and ST164 mediates dissemination of the *bla*_NDM-1_/*bla*_OXA-23_ profile in *Acinetobacter baumannii*

**DOI:** 10.1186/s12941-026-00872-5

**Published:** 2026-06-22

**Authors:** Ren Liu, Guilin Jin, Feng Yu, Hui Tan, Fang Yuan, Peiwen Zhang, Yinyi Chen, Liang Xiao, Xiaojun Yang

**Affiliations:** Department of Clinical Laboratory, The Affiliated Hospital of Jiangxi University of Chinese Medicine, Jiangxi University of Chinese Medicine, 445 Bayi Avenue, Nanchang, 330006 Jiangxi People’s Republic of China

**Keywords:** *Acinetobacter baumannii*, *Bla*_NDM-1_, *Bla*_OXA-23_, Clonal expansion, Phylogenomics

## Abstract

**Background:**

The convergence of the potent carbapenemase genes *bla*_NDM-1_ and *bla*_OXA-23_ in carbapenem-resistant *Acinetobacter baumannii* (CRAB) poses a critical threat. However, the dissemination patterns and evolutionary drivers of this dual-carbapenemase profile remain unclear.

**Methods:**

We performed a comprehensive genomic analysis of a global collection of 1820 high-quality public WGS-derived CRAB isolates (2001–2024) harbouring acquired non-OXA carbapenemase genes. Topological congruence between *bla*_NDM-1_- and *bla*_OXA-23_-positive phylogenies was assessed via normalized Robinson-Foulds (nRF) distance. Ecological diversity metrics (e.g., Shannon entropy) combined with Bayesian and hypergeometric models quantified host-lineage restriction. The evolutionary dynamics of a colistin-resistant ST2 sublineage (PmrB T232I) were inferred using Skygrowth coalescent analysis. Geographic divergence was assessed via PERMANOVA and genome-wide association study (GWAS).

**Results:**

Co-occurring acquired carbapenemase genes dominated the cohort, with the combination of *bla*_NDM_-type and *bla*_OXA-23_-like genes accounting for 64.7% (1178/1820) of isolates. This prevalence was primarily driven by the specific *bla*_NDM-1_/*bla*_OXA-23_ profile (*n* = 1080), overwhelmingly restricted to two clones: the internationally disseminated ST2 (*n* = 644) and the regionally prevalent ST164 (*n* = 209). An integrated triad of evidence—topological congruence (nRF = 0.346, *p* < 0.001), a mathematically stark collapse in lineage diversity upon dual-gene acquisition, and extreme statistical enrichment (Bayes factors 7.8 × 10^19^–1.5 × 10^22^)—demonstrates that this dual-resistance phenotype does not diffuse randomly via horizontal transfer. Instead, it is profoundly constrained within these specific accommodating clonal frameworks. Within the dominant ST2 clone, a colistin-resistant high-risk sublineage exhibited a ‘gene load–selection continuum’, where a subgroup with an expanded resistome paradoxically maintained a higher effective population size, highlighting the role of genomic plasticity. Geographic origin explained 46.5% of accessory genome variance (PERMANOVA *p* < 0.001), highlighting strong geographic stratification of accessory genomes.

**Conclusions:**

The widespread dissemination of the *bla*_NDM-1_/*bla*_OXA-23_ profile is mediated by the convergent expansion of ST2 and ST164, acting as dominant clonal drivers. This study provides a framework for clone-specific epidemiological hitchhiking and highlights the need to refine surveillance to prioritize the genomic tracking of these key lineages. The retrospective nature of public data may influence prevalence estimates, but the core clonal dissemination pattern remains robust.

**Supplementary Information:**

The online version contains supplementary material available at https://doi.org/10.1186/s12941-026-00872-5.

## Introduction

Antimicrobial resistance (AMR) is a significant global health concern, causing approximately 1.27 million deaths annually [1]. By 2050, this number could exceed 10 million, surpassing diabetes and cancer [2]. Carbapenem antibiotics are effective against most beta (β)-lactamase-producing bacteria. The increased proliferation of carbapenem-resistant Enterobacteriaceae (CRE) [3] and *Acinetobacter baumannii* (CRAB) [4] limits this treatment option. CRAB, in particular, has emerged as a formidable nosocomial pathogen since its identification in the 1970s, notorious for its extreme drug resistance [5].

The World Health Organization (WHO) classifies CRAB as a critical-priority pathogen [6]. Global surveillance data paint an alarming picture: the rate of carbapenem-nonsusceptible *A. baumannii* in intensive care units (ICUs) has surged from ≈ 30% in 2005 to > 80% in 2023 [7, 8]. These infections disproportionately affect immunocompromised and elderly ICU patients, incurring mortality rates of 22.8%–71.6% and imposing billions in annual healthcare costs [9, 10].

The genetic basis of carbapenem resistance in *A. baumannii* is multifactorial, involving alterations in drug targets, porins, and efflux pumps [11]. Yet, the acquisition of carbapenemase genes represents the dominant and most consequential mechanism [12]. Among these, acquired Class D β-lactamases [oxacillinases (OXAs)] are paramount. Specifically, *bla*_OXA-23_-like has become the predominant global determinant [13, 14], its pandemic success fueled by stable integration into widespread, IS*Aba1*-bounded composite transposons like Tn*2006*, where functionally active IS*Aba1* drives both transposition and enhanced gene expression [15], enabling chromosomal fixation and clonal spread [13, 16]. Other acquired OXA genes (e.g., *bla*_OXA-58_-like, *bla*_OXA-24/40_-like) drive regional epidemics. In contrast, the intrinsic chromosomal *bla*_OXA-51_-like genes confer minimal resistance unless overexpressed by an upstream insertion sequence (e.g., IS*Aba1*) [12, 17, 18]. Class B metallo-β-lactamases (MBLs) are potent carbapenemases. Among these, the New Delhi MBL (NDM) is of particular concern; the genes encoding these enzymes (e.g., *bla*_NDM_) are reported less frequently in *A. baumannii* but constitute an emerging threat, often linked to plasmid-mediated transmission [13, 16].

The remarkable genomic plasticity of *A. baumannii* [19]—manifesting as a highly dynamic, promiscuous resistome driven by horizontal gene transfer and abundant mobile elements [20, 21]—coupled with its capacity for healthcare-associated transmission [22], has given rise to a complex global epidemiology. The distribution of CRAB clones and their associated carbapenemase genes exhibits marked geographic variation [23]. While *bla*_OXA-23_ has been the focal point of routine surveillance, strains harboring potent Ambler Class A or B carbapenemase genes (e.g., *bla*_NDM_)—either alone or, more worryingly, in combination with acquired OXA genes—represent an escalating treatment crisis due to the severe lack of effective clinical inhibitors. Despite this, a systematic, population-genomic investigation dedicated to this specific sub-population of CRAB isolates harboring one or more acquired non-OXA carbapenemase genes is conspicuously absent. This critical knowledge gap impedes our understanding of the evolutionary drivers and dissemination networks behind these most intractable forms of CRAB.

To address this, we conducted a comprehensive genomic analysis of all available *A. baumannii* genomes in the National Center for Biotechnology Information (NCBI) database, culminating in a curated global cohort of 1,820 CRAB isolates (2001–2024) defined by the presence of at least one acquired non-OXA carbapenemase gene. Within this specific cohort, our analysis placed particular emphasis on dual-carbapenemase carriers. Our study aimed to (i) delineate the global dissemination routes of the ST2 and ST164 clones, which emerged as the dominant lineages specifically among isolates co-harboring *bla*_NDM_-type and *bla*_OXA-23_; (ii) quantify the gene-load/selection-fitness dynamics underpinning their expansion; and (iii) synthesize the evidence into a coherent model of clone-specific epidemiological hitchhiking. Our findings provide actionable insights for shifting surveillance paradigms and targeting containment strategies against these dual-carbapenemase-producing clones.

## Materials and methods

### Compilation, curation, and basic characterization of the specific CRAB genomic cohort

A total of 28,776 *A. baumannii* genomic assemblies were initially retrieved from the NCBI database (accessed July 2024). To ensure data integrity while preserving the collection’s temporal span (2001–2024) and epidemiological representativeness, we applied a tiered quality control pipeline. First, assemblies with excessive fragmentation (contig count > 1000) were excluded to minimize assembly errors [24]. Second, species identity was stringently confirmed through Average Nucleotide Identity (ANI) analysis against the *A. baumannii* reference genome (GCF_008632635.1) using FastANI v1.33 [25], retaining only isolates with ≥ 95% ANI [26]. Finally, genomic completeness and contamination were assessed using CheckM v1.1.10 [27]. CheckM thresholds were initially adjusted to inclusive standards tailored for large-scale longitudinal surveillance (Completeness ≥ 90% and Contamination ≤ 10%) [27], accounting for biological characteristics such as frequent insertion sequences (ISs). However, to avoid discarding biologically significant historical isolates displaying inflated contamination estimates due to strain heterogeneity (common in clinical *A. baumannii*), we applied a secondary rescue step. Genomes exceeding the 10% contamination limit were further scrutinized using GUNC v1.0.5 [28]. Genomes displaying lineage homogeneity (Clade Separation Score [CSS] < 0.45) were classified as having redundant but non-chimeric content and were retained, as they provide reliable signals for core-genome phylogenetics. This strategy resulted in a robust genomic dataset for subsequent resistance screening.

From this quality-controlled set, antibiotic resistance genes (ARGs) were screened via the Comprehensive Antibiotic Resistance Database (CARD) [29]. To ensure high-specificity detection, we applied a stringent threshold: hits were retained only if they simultaneously satisfied nucleotide identity ≥ 80%, alignment coverage ≥ 80%, and a combined identity × coverage product ≥ 0.8. This criterion is more conservative than that used in many large-scale resistance gene surveys (e.g., ≥ 90% identity and ≥ 60% coverage [23]). This study specifically focused on isolates carrying acquired non-OXA carbapenemase genes (e.g., *bla*_GES_, *bla*_KPC_, *bla*_NDM_, *bla*_IMP_, *bla*_VIM_). Based on this inclusion criterion, we defined a final cohort of 1820 CRAB isolates harboring at least one of these non-OXA genes, regardless of the co-presence of OXA-type genes (Table S1). It is important to clarify that isolates carrying solely acquired OXA-type carbapenemase genes (e.g., *bla*_OXA-23_ only) were not selected for this specific cohort. This selection strategy allowed us to investigate the molecular epidemiology of the increasingly challenging Ambler Class A and B carbapenemase-producing *A. baumannii*, which pose severe clinical threats due to the lack of highly effective inhibitors, particularly for the MBLs [30].

All 1,820 isolates were thereafter subjected to fundamental genomic characterization. Multilocus sequence typing (MLST) was conducted following the Pasteur scheme [31]. International clone (IC) assignments were derived directly from the MLST results based on established ST-IC correlations [12, 32–34]. For sequence types (STs) that could not be assigned to a defined IC, a BURST analysis was employed to cluster them into clonal complexes (CCs) [35]. Additionally, PmrA and PmrB sequences were aligned against the widely used reference strain *A. baumannii* ATCC 17978 to identify mutations [36, 37]. From this comprehensively characterized cohort, a specific colistin-resistant ST2 sublineage (*n* = 175), primarily defined by the prevalent PmrB T232I mutation, was identified. Given the severe therapeutic dead-end posed by concurrent resistance to carbapenems and colistin (a last-resort antibiotic), this group was conceptually defined as a "colistin-resistant high-risk sublineage" for the purpose of this study, and served as the focal group for subsequent evolutionary analysis.

### Bioinformatics analysis for phylogenetic and specific element screening

Genomic annotation was carried out using Prokka version 12-beta [38]. Core genes were identified using Roary v3.13.0 [39], with a threshold of 95% identity for clustering and a core gene definition of 99% strain prevalence, forming the basis for all phylogenetic reconstructions. Subsequently, specific genetic elements were characterized. ISs were identified using the ISfinder database [40]. Plasmid replicon typing was performed using the Acinetobacter Typing database [41]. Capsular polysaccharide (K locus, KL) [42] and lipooligosaccharide (OC locus, OCL) loci [43] were analyzed using Kaptive. Furthermore, driven by recent reports implicating prophages in the horizontal transfer of carbapenemases [44, 45], we systematically screened the entire cohort (*n* = 1820) for prophage sequences using VIBRANT v1.2.0 [46]. The predicted prophage coordinates were subsequently intersected with the genomic positions of *bla*_NDM-1_ and *bla*_OXA-23_ to assess prophage-mediated carriage of these specific resistance determinants.

### Phylogenetic analysis

#### Core genome phylogenetic reconstruction

A maximum-likelihood (ML) phylogeny was inferred from a core genome alignment of 1,245 single-copy orthologues across all 1,820 isolates using FastTree 2.1.11 [47]. Given the high conservation of these single-copy orthologues within this closely related species complex, tree reconstruction was performed using the default Jukes-Cantor (JC) evolutionary model combined with the CAT approximation to account for varying rates of mutation across sites. Following this, focused trees for the dominant ST2 clone (*n* = 781) were constructed. To quantitatively define thresholds for distinguishing ICs, pairwise single-nucleotide polymorphism (SNP) distances were calculated from the core genome alignment. The optimal SNP threshold for cluster separation was determined by maximizing the silhouette index; isolates with pairwise SNP differences ≤ 100 were considered potentially clonal or closely related, in line with established criteria [14]. The distribution of pairwise distances and the silhouette analysis curve were visualized using ggplot2.

#### Country-specific and gene-specific phylogenies

Separate ML phylogenies for *bla*_NDM-1_ (*n* = 1032) and *bla*_OXA-23_ (*n* = 962) were built from the isolates from the top three countries (the USA, China, and India), based on the same core genome alignment.

### Assessment of non-random clonal restriction via tree topology

Prior to comparison, both the *bla*_NDM-1_ and *bla*_OXA-23_ phylogenies were pruned to include only the 909 isolates common to both datasets using the keep.tip function from the R package ape. Furthermore, subtrees were extracted for ST2 (*n* = 638) and ST164 (*n* = 209) isolates to assess clone-specific trends. All trees contained multifurcations, which were retained to avoid bias from arbitrary binary resolution. Topological distances, including the normalized Robinson-Foulds (nRF) distance, the Kendall-Colijn (KC) distance, and the Branch Score Difference (BSD), were calculated using the TreeDist v2.10.1 package [48]. The statistical significance of the observed distances was assessed using a permutation test (1000 replicates). To explicitly avoid circular reasoning regarding gene co-evolution and to test for non-random clonal restriction, the null hypothesis posited that the dual-carriage of these genes is entirely independent of the underlying core-genome population structure. Accordingly, this test preserved the topology of the *bla*_OXA-23_ tree while randomly reshuffling the tip labels, allowing us to statistically confirm whether the observed phylogenetic congruence reflects a tight, non-random association with specific clonal lineages (e.g., ST2 and ST164) rather than chance distribution.

### Population diversity and clonal restriction metrics

To quantitatively assess the genetic bottlenecking and host restriction of the dual-carbapenemase profile, ecological diversity indices were evaluated based on the ST distribution of the isolates. The Shannon–Wiener index (*H*′) [49], Simpson's index of diversity (1 − *D*) [26], and Pielou's Evenness (*J*′) were calculated for cohorts carrying *bla*_NDM-1_ alone, *bla*_OXA-23_ alone, and the *bla*_NDM-1_ + *bla*_OXA-23_ combination. Calculations were performed using the vegan package (v2.6-4) in R. In this context, the Shannon entropy translates to the effective diversity of host genetic backgrounds, where a marked reduction in *H*′ and Evenness signifies profound ecological restriction (extreme enrichment within specific clonal lineages) rather than stochastic, widespread dissemination.

### Bayesian evolutionary analysis

To investigate the evolutionary dynamics of the colistin-resistant ST2 sublineage (*n* = 175, defined by PmrB T232I mutation), a time-scaled phylogeny was reconstructed. Temporal signal was confirmed using a root-to-tip regression in TempEst v1.5.3 [50] (*R*^2^ = 0.363). The Bayesian analysis was performed in BEAST v1.10.4 [51] using a gamma site model and a strict clock model. The Markov Chain Monte Carlo (MCMC) chain was run for 300 million generations, with the first 10% discarded as burn-in. The time to the most recent common ancestor (*t*MRCA) and evolutionary rate were estimated. Statistical uncertainty was assessed using 95% highest posterior density (HPD) intervals. The effective population size (*Ne*) through time was estimated from the posterior tree distribution using the skygrowth method in R [52], running the MCMC for 200,000 steps with a thinning interval of 100 and a smoothing parameter (tau0) of 0.1. Model convergence was confirmed, with all parameters achieving effective sample size (ESS) values > 789.

### Association analysis between gene load and effective population size

Within the colistin-resistant ST2 sublineage (*n* = 175), isolates were categorized into the 'Core Dual-Carbapenemase Group' (*n* = 158, *bla*_NDM-1_ and *bla*_OXA-23_ only) and the 'Extended Resistome Group' (*n* = 17, carrying additional carbapenemase genes). Specifically, 19 genomes within the 'Core Dual-Carbapenemase Group' exhibited assembly redundancy (validated as lineage-homogeneous by GUNC). We retained these genomes in the baseline group based on the rationale that redundancy-associated inflation of gene counts or genetic diversity would only introduce a conservative bias (i.e., reducing the magnitude of difference between this baseline group and the high-load 'Extended Resistome Group'). Thus, significant differences observed despite this inclusion are considered robust. Differences in ARG counts, plasmid replicon counts, IS counts, and *Ne* between the two groups were assessed using the two-sided Wilcoxon rank-sum test. To control the false discovery rate, *p*-values from the three pairwise comparisons (ARGs, plasmids, ISs) were adjusted using the Benjamini-Hochberg method (FDR ≤ 0.05); the comparison of *Ne* was performed separately and is reported with its nominal *p*-value. To explore the relationship between *Ne* and the load of mobile genetic elements, we fitted second-order polynomial (quadratic) regression models between *Ne* and the numbers of acquired ARGs, ISs, and plasmid replicons. For each model, the adjusted *R*^2^ and the *p*-value from the *F*-test are reported. Given the exploratory nature of these regression analyses, *p*-values were not adjusted for multiple comparisons. All analyses were conducted in R v4.5.1.

All phylogenetic trees were visualized using tvBOT [53].

### Clonal association and distribution of the dual-carbapenemase backbone

The observed phylogenetic congruence between *bla*_NDM-1_ and *bla*_OXA-23_ prompted the hypothesis of lineage-associated preference. To test this against the null model of random assortment, we analyzed the co-occurrence of the second carbapenemase in *bla*_NDM-1_ carriers (*n* = 1032) and *bla*_OXA-23_ carriers (*n* = 962), stratified by ST2, ST164, and Other clones.

(i) Co-occurrence significance analysis: Clonal enrichment was evaluated using an integrated framework. Pearson’s χ^2^ test assessed the global association. Pairwise comparisons were conducted using two-tailed Fisher’s exact tests on continuity-corrected tables (adding 0.5 to all cells when any zero was present), reporting odds ratios with exact 95% confidence intervals. Bayes factors were computed under a Dirichlet-multinomial model. The significance of observed co-occurrence rates was evaluated by one-tailed tail-area probability across 10,000 hypergeometric replicates under the null model. All *p*-values were Benjamini–Hochberg adjusted (FDR ≤ 0.05).
(ii) Accessory-gene association: An accessory gene enrichment analysis was performed on all isolates from the three countries (*n* = 1104), including ARGs, ISs, and plasmid replicons. Features were filtered by prevalence (≥ 20% in any ST group) or statistical significance (*p* < 0.05 with |log₂OR|≥ 2).
(iii) Genetic context and plasmid mobility analysis: The genetic environments of *bla*_NDM-1_ and *bla*_OXA-23_ were analyzed using a set of complete genomes (n = 46), with a focus on but not limited to ST2 and ST164 lineages. Flanking regions were delineated using NCBI.gff annotations and SnapGene v8.0, with visualizations created in Chiplot [53]. Subsequently, the genomic localization of all carbapenemase genes was determined, and plasmid mobility was predicted based on the presence of MOB and T4SS genes (mobilizable: MOB+; conjugative: MOB+ and T4SS+) [54].

### Analysis of geographic stratification within dominant clones

Building on the evidence of clone-driven dissemination, we investigated whether the dominant ST2 and ST164 clones exhibited geographic substructure in their accessory genome profiles. First, a three-dimensional Bray–Curtis principal coordinates analysis (PCoA) was performed on the combined ARG-IS-plasmid presence–absence matrix of the same USA, China and India isolate set (*n* = 1104) used in the clone-specific analyses. Significance of country-level differentiation was then evaluated with PERMANOVA (adonis2, 9999 permutations) under three sequential models: (i) country alone; (ii) country with ST type (ST2/ST164/Others) as additive covariate; and (iii) full country × ST interaction.

A genome-wide association study (GWAS) was conducted using Scoary [55] with the core-genome phylogeny included to correct for population structure. Significantly associated genes (FDR < 0.05) were subjected to KEGG pathway enrichment analysis [56].

### Statistical analysis

All statistical analyses and data visualizations (including violin plots, bubble charts, heatmaps and GWAS Manhattan plots) were performed in R v4.5.1. The Benjamini–Hochberg procedure was applied throughout the study to control the FDR at 0.05, unless otherwise specified in the respective method subsections.

## Results

### The global ascendancy of co-harbored *bla*_NDM-1_ and *bla*_OXA-23_ among non-OXA carbapenemase-producing CRAB is mediated by clonal expansion of the ST2 lineage

To delineate the drivers behind the global spread of this specific sub-population—CRAB harboring at least one acquired non-OXA carbapenemase gene—we analyzed a curated global cohort of 1820 isolates (Table S1). To further contextualize the ecological distribution of this cohort, we categorized the available isolation sources. Consistent with prevailing trends in public genomic repositories, we observed a profound sampling bias toward human healthcare settings: 1710 isolates (94.0%) were of clinical origin. Non-clinical origins were markedly less represented, comprising environmental (*n* = 17) and animal (*n* = 2) sources, alongside laboratory (*n* = 4) and unknown (*n* = 87) backgrounds. Despite the limited non-clinical representation, this rich global dataset provides a robust foundation for genomic epidemiological analysis. Beyond the defining carbapenemase genes, this cohort exhibited a rich landscape of antimicrobial resistance determinants. Nearly all isolates harbored genes conferring resistance to aminoglycosides and fluoroquinolones (> 98%), with a high prevalence of macrolide resistance genes (68.3%) also observed. Notably, 28 isolates carried the tigecycline resistance gene *tet*(X), underscoring the emergent threat to last-resort therapies.

A striking finding was the predominance of co-occurring acquired carbapenemase genes, observed in 1426 (78.4%) isolates (Table S2). Among these, the combination of *bla*_NDM_-type and *bla*_OXA-23_-like genes was overwhelmingly dominant, accounting for 64.7% (*n* = 1178) of the entire cohort. Specifically, the *bla*_NDM-1_ and *bla*_OXA-23_ dual-carbapenemase gene profile represented the most prevalent combination (*n* = 1080), establishing itself as the central focus of this study. Notably, within our specifically curated cohort, this highly successful *bla*_NDM-1_/*bla*_OXA-23_ combination was exclusively identified in isolates of clinical and limited environmental origins. It was entirely absent in the two available animal-derived genomes, which instead harbored *bla*_NDM-1_ alone and a *bla*_NDM-1_/*bla*_OXA-58_ combination, respectively.

**Fig. 1:**
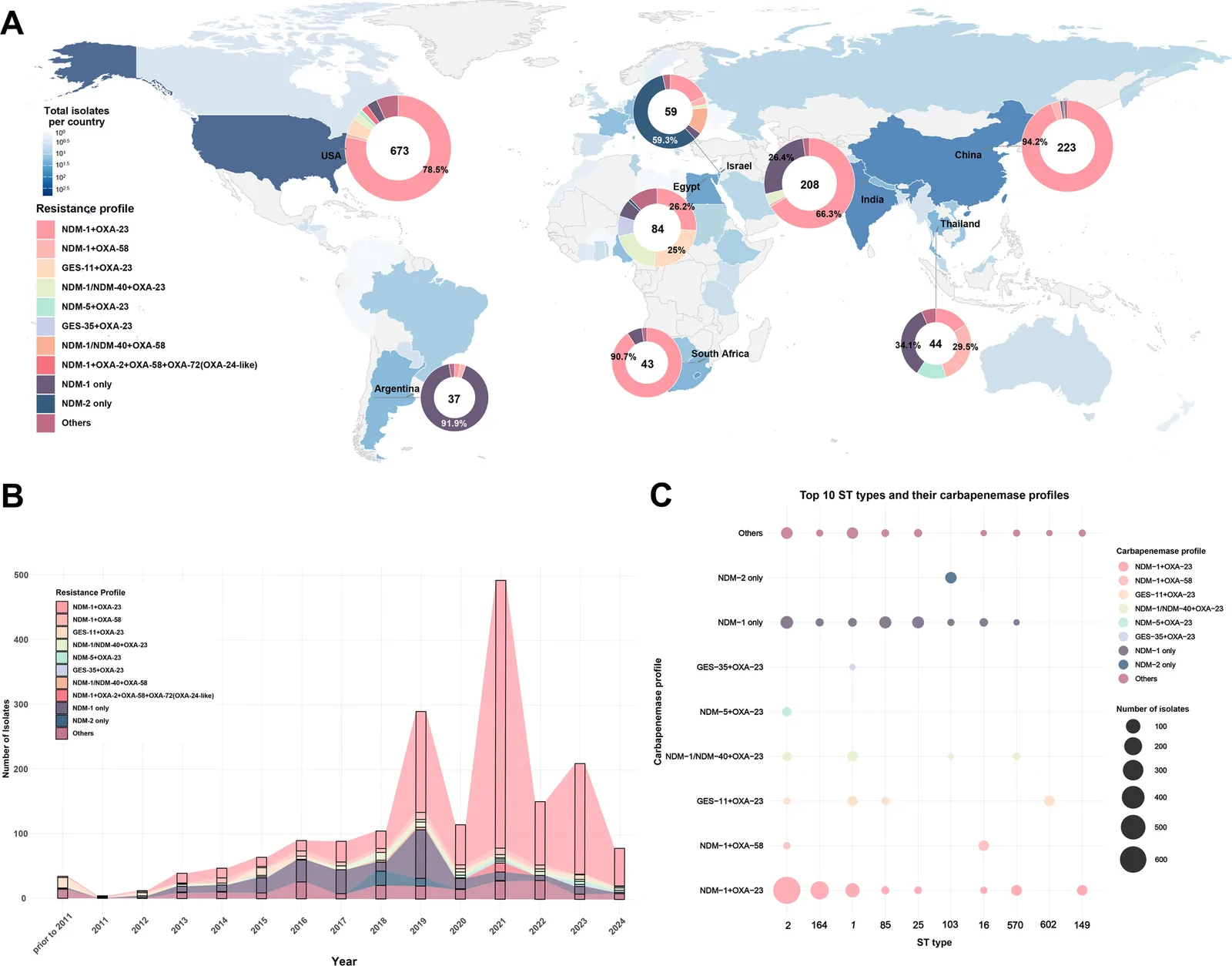
Global dissemination and clonal architecture of dual-carbapenemase CRAB. (In these figures, carbapenemase genotypes are represented by the corresponding enzyme names for visual clarity.). **A** Worldwide distribution and national composition. Country colour intensity indicates the total isolate number. Doughnut charts for the top eight countries show the proportional composition of the eleven resistance profiles; major combinations (e.g., NDM-1 + OXA-23) are labelled, with less frequent profiles pooled as “Others”. **B** Temporal dynamics of major dual-carbapenemase patterns. Stacked-area plot of annual isolate counts (y-axis) from pre-2011 to 2024 (x-axis). Coloured bands represent the most prevalent enzyme combinations; rarer patterns are aggregated into “Others”. **C** Clone–enzyme associations. Bubble plot of the top 10 STs (x-axis) versus primary enzyme combinations (y-axis). Bubble area scales with isolate number and is coloured by profile; ST2 is overwhelmingly associated with NDM-1 + OXA-23

An analysis of the geographic distribution within our curated cohort highlights the widespread global dissemination of these dual-carbapenemase profiles (Fig. 1A). (In figures, dual-carbapenemase profiles are denoted using enzyme names [e.g., NDM-1 + OXA-23] for visual clarity.) Among the top three countries by isolate count—the United States (37.0%, 673/1820), China (12.3%, 223/1820), and India (11.4%, 208/1820)—the co-occurrence of *bla*_NDM-1_ and *bla*_OXA-23_ emerged as the predominant genotype within this targeted sub-population, underscoring its international success. Notably, this particular combination constituted 78.5% (528/673) of the analyzed U.S. isolates, 94.2% (210/223) of the Chinese isolates included in our study, and 66.3% (138/208) of the Indian cohort. In contrast, other countries exhibited different dominant profiles among their non-OXA isolates, such as the prevalence of *bla*_NDM-1_ only in Thailand (34.1%, 15/44) and Argentina (91.9%, 34/37), and *bla*_NDM-2_ only in Israel (59.3%, 35/59), indicating distinct regional epidemics.

Temporal analysis revealed the rapid expansion of isolates co-harboring *bla*_NDM-1_ and *bla*_OXA-23_ (Fig. 1B). First identified in 2010 in two Egyptian isolates, the frequency of this dual-carriage profile increased markedly from 2019 onwards, quickly establishing itself as a dominant force within this global non-OXA carbapenemase-producing sub-population. This recent surge suggests the acquisition of factors conferring a significant evolutionary advantage.

We next investigated the clonal background associated with this combination (focusing on the top 10 most prevalent STs). A striking clonal predominance was observed for specific carbapenemase profiles (Fig. 1C). Among these isolates co-harboring *bla*_NDM-1_ and *bla*_OXA-23_, the IC2 (ST2) clone was the predominant vehicle, accounting for 63.7% (644/1011) of such strains. ST164 emerged as the second most significant clone, accounting for 20.7% (209/1011) of isolates co-harboring *bla*_NDM-1_ and *bla*_OXA-23_. Other profiles were associated with specialized clones, such as ST16 with *bla*_NDM-1_ and *bla*_OXA-58_ (70.3%, 26/37), ST103 with *bla*_NDM-2_ only (92.9%, 39/42), and ST602 with *bla*_GES-11_ and *bla*_OXA-23_ (96.6%, 28/29).

The temporal synergy between the expansion of the ST2 clone and the *bla*_NDM-1_ and *bla*_OXA-23_ gene profile was unequivocally demonstrated (Fig. S1A). The population-level increase of isolates carrying *bla*_NDM-1_ and *bla*_OXA-23_ after 2019 was paralleled by a concurrent rise in ST2 isolates carrying this dual-gene profile. Furthermore, analysis of the ST2 lineage's internal evolutionary trajectory revealed that this *bla*_NDM-1_ and *bla*_OXA-23_ profile, first detected within ST2 in a 2014 isolate from Nepal, began its multinational expansion in 2017, with early outbreaks notably centered in India (Fig. S1B).

In summary, these findings reveal that the prominence of the *bla*_NDM-1_/*bla*_OXA-23_ combination among globally distributed non-OXA CRAB is primarily mediated by the expansion of the ST2 clone, with a substantial secondary contribution from ST164. This clonal success establishes ST2 and ST164 as the key epidemiological vectors driving the widespread dissemination of this highly resistant genotype.

### Population genomics unveils a colistin-resistant high-risk sublineage within the widespread ST2 clone

**Fig. 2:**
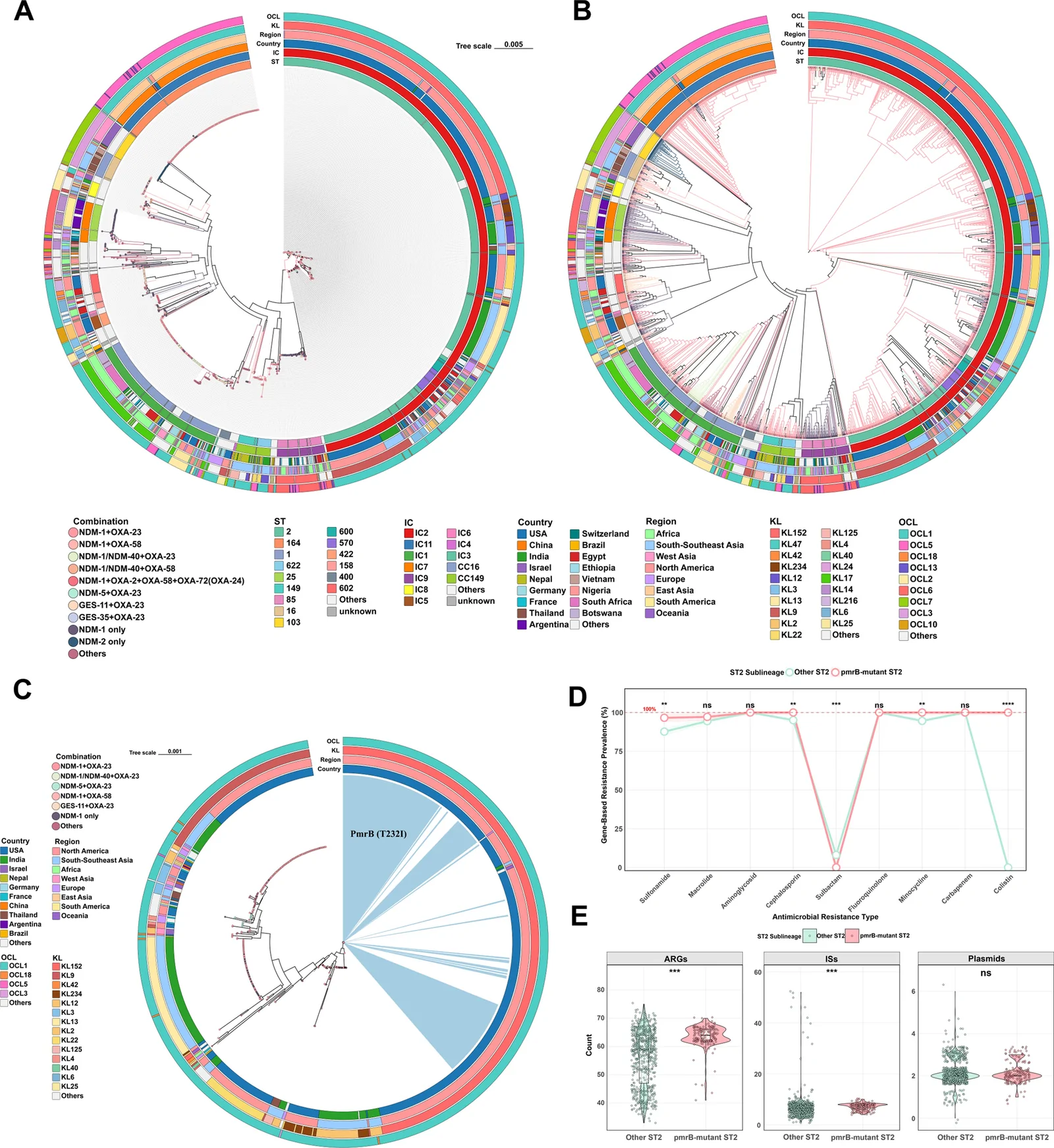
Population genomics of 1,820 CRAB isolates and the colistin-resistant high-risk ST2 sublineage. (In these figures, carbapenemase genotypes are represented by the corresponding enzyme names for visual clarity.) **A** Maximum-likelihood phylogeny with branch lengths proportional to substitutions per site. **B** Equal-branch cladogram of the same alignment highlighting topological structure. Rings (inner → outer): ST, IC, country, continent, KL, OCL. Tip colors correspond to Fig. 1. **C** Focused phylogeny of the ST2 lineage (*n* = 781). Rings: country, continent, KL, OCL. Blue sector highlights the 175 PmrB-T232I isolates (all KL152/OCL1). **D** Comparative resistance phenotypes. Lines show gene-based resistance prevalence (%) across nine drug classes between “PmrB-mutant ST2” (*n* = 175) and “Other ST2” (*n* = 606). **p < 0.01, ***p < 0.001 (χ^2^ test). **E** Accessory genome burden. Violin plots compare ARG, IS, and plasmid replicon counts. The PmrB-mutant sublineage carries significantly more ARGs and ISs. Statistical significance was assessed using the Wilcoxon rank-sum test, and *p*-values were adjusted for multiple comparisons using the Benjamini–Hochberg procedure. ***p < 0.001; ns, not significant

To delineate the population structure underlying the widespread dissemination of this targeted non-OXA CRAB cohort, we reconstructed a core-genome ML phylogeny of the 1,820 isolates. We present this phylogeny in two complementary views to fully resolve both evolutionary dynamics and topological relationships (Fig. 2A, B). The branch-length tree (Fig. 2A) revealed that the major ST2 lineage exhibited exceptionally short internal branch lengths, a phylogenetic signature indicative of a recent, rapid multinational expansion. This compressed structure, however, obscured the fine-scale topology. The equal-branch tree (Fig. 2B) unequivocally demonstrated that the predominant genetic profile within our study population—co-harboring *bla*_NDM-1_ and *bla*_OXA-23_—was not randomly distributed but displayed a high degree of phylogenetic clustering within specific clades, primarily ST2 and ST164.

While the combination of *bla*_NDM-1_ and *bla*_OXA-23_ was identified across 21 different STs from 35 countries, confirming its broad dissemination potential, its population-level success was demonstrably clonal. ST2 and ST164 together accounted for 79.0% of all isolates carrying these two genes. This clonal dominance among *bla*_NDM-1_ and *bla*_OXA-23_ co-harboring isolates was further corroborated by distinct KL and OCL backgrounds: ST2 isolates carrying this combination were predominantly KL152/OCL1 (55.3%), whereas their ST164 counterparts were almost exclusively KL47/OCL5 (96.7%).

Quantitative analysis firmly established the genetic boundaries between these successful lineages. Calculation of pairwise SNP distances from the core genome alignment determined an optimal threshold of 95 SNPs for cluster separation (maximum silhouette index = 0.928), with the minimum distance between any two International Clones (e.g., IC2 vs. IC1) being 6,637 SNPs (Fig. S2A). This confirms the deep phylogenetic divergence between the major circulating clones.

We next asked what distinguished the hyper-successful ST2 clone from other prevalent STs. Comparative analysis of accessory genome elements revealed that ST2 isolates carried a significantly higher burden of ARGs compared to other major STs (Median: 61 vs. 34–54.5, *p* < 0.001; Fig. S2B), highlighting its extreme multidrug-resistant phenotype. While its plasmid replicon count was not the highest (Median: 2 vs. 2–3 in other STs), it maintained a significant IS load (Median: 7 vs. 2–14 across other STs), suggesting a genetically active background permissive to the acquisition and rearrangement of resistance determinants.

A Sublineage with Pan-Drug Resistant Potential Emerges within ST2. To systematically identify determinants of colistin resistance, we profiled mutations in the PmrAB two-component system across the entire collection [57]. While over 90% of isolates had a wild-type PmrA sequence, 99.7% harbored at least one non-synonymous mutation in PmrB, with 64 distinct substitutions identified across the protein (Fig. S2C). From this background of extensive variation, a single mutation of paramount concern emerged: the T232I substitution, located adjacent to the conserved histidine kinase core (H228) and previously linked to colistin resistance [58, 59].

Within the widespread ST2 clone, a subpopulation of utmost clinical concern emerged: the previously defined "Colistin-Resistant High-Risk ST2 Sublineage" (*n* = 175), characterized by the PmrB T232I mutation. A focused phylogenetic analysis clearly demarcated this high-risk sublineage, which was exclusively associated with the KL152/OCL1 background (Fig. 2C). These mutant isolates exhibited two key phylogenetic features: very short branch lengths and a star-like topology, a pattern consistent with a recent and rapid clonal expansion.

Phenotypic profiling confirmed the grave threat posed by this sublineage. Compared to other ST2 isolates, the PmrB-mutant ST2 group exhibited significantly higher resistance rates to multiple last-line and cornerstone antibiotics, including cephalosporins, minocycline, and colistin (*p* < 0.01; Fig. 2D), indicating a progression toward pan-drug resistance.

Strikingly, this high-risk sublineage was also genetically distinct, carrying a significantly heavier burden of both ARGs and ISs compared to other ST2 isolates (ARGs Median: 64 vs. 59, *p* < 0.001; ISs Median: 8 vs. 6, *p* < 0.001; Fig. 2E). This indicates that the evolution of colistin resistance has occurred within a genetic background that is not only hyper-resistant but also continues to accumulate additional resistance genes, likely facilitated by a more active mobilome.

### Evolutionary dynamics of the colistin-resistant high-risk ST2 sublineage

**Fig. 3:**
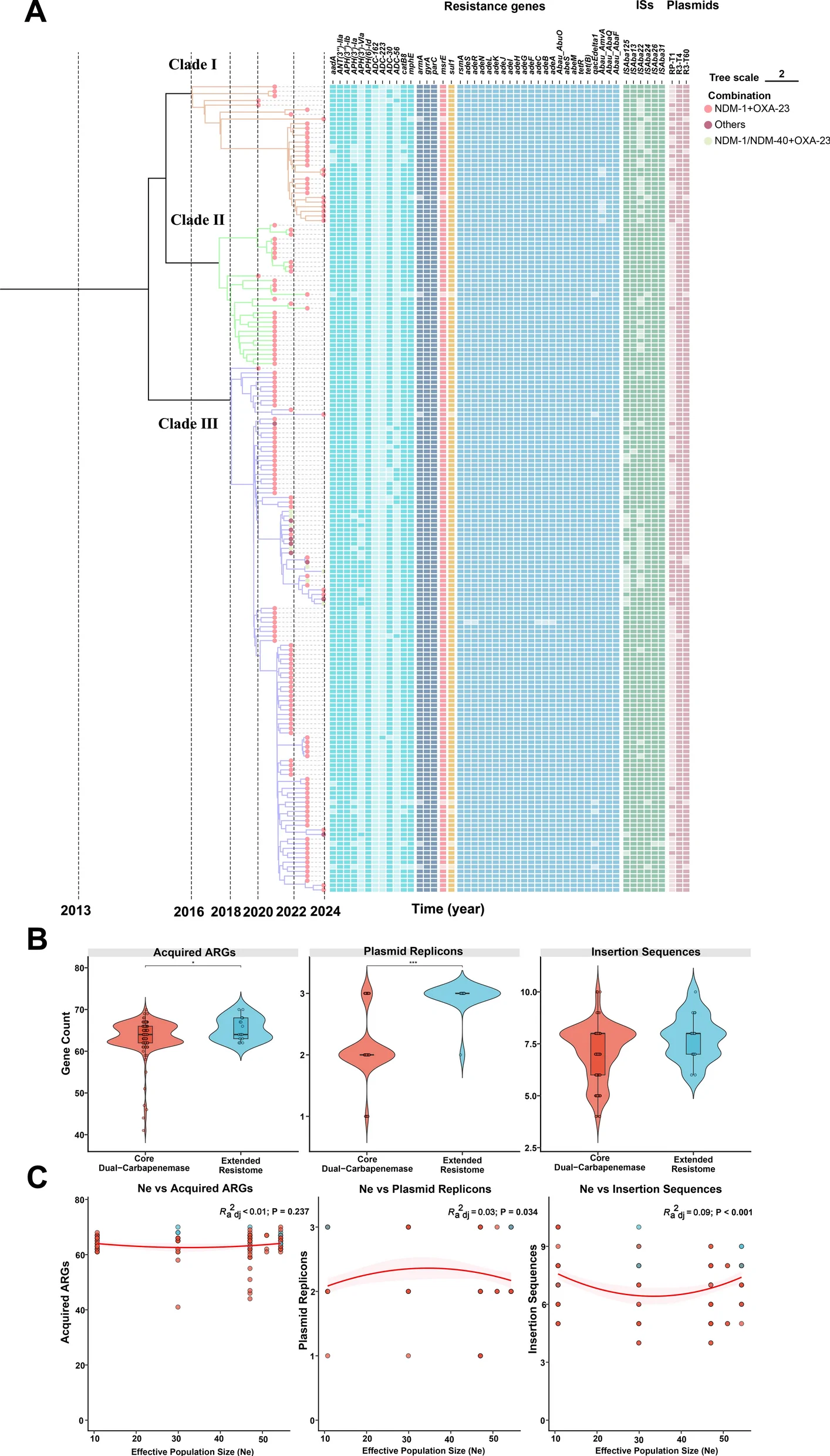
Evolutionary dynamics and population history of the colistin-resistant high-risk ST2 sublineage. **A** Time-calibrated Bayesian phylogeny of the 175-isolate PmrB-T232I sublineage, reconstructed using BEAST. The rectangular tree highlights major Clades (I-III). Metadata columns (right) display profiles of ARGs, ISs, and plasmid replicons for each isolate. **B** Comparative genomic analysis of ST2 subgroups. Violin plots compare the distribution of ARG, IS, and plasmid replicon counts between the “Core Dual-Carbapenemase Group” (*n* = 158) and the “Extended Resistome Group” (*n* = 17). Comparisons of effective population size (*Ne*) are shown in Fig. S3B. *p < 0.05, ***p < 0.001 (Wilcoxon rank-sum test with Benjamini–Hochberg correction). **C** Relationship between mobile genetic elements and effective population size. Scatter plots show *Ne* against counts of acquired ARGs, plasmid replicons, and IS elements. The adjusted *R*^2^ and *p*-values derived from quadratic polynomial regression models are reported for specific associations

Building on the phylogenetic and genomic insights from the previous sections, we conducted a Bayesian dating analysis of the 175-isolate PmrB-T232I sublineage. The time-calibrated tree (Fig. 3A) placed *t*MRCA of this high-risk group around 2013 (95% HPD: 2005–2018), with a mean substitution rate of 2.6 × 10⁻⁷ substitutions/site/year (95% HPD: 1.5 × 10⁻⁷–4.9 × 10⁻⁷). This timescale aligns closely with the global increase in colistin use for treating CRAB infections [60].

The skyline plot (Fig. S3A) revealed a dynamic population trajectory: rapid expansion began around 2019 (coinciding with heightened CRAB surveillance during the COVID-19 pandemic), followed by a temporary bottleneck in 2021 and a subsequent recovery. Despite low genetic diversity (*Ne* ~ 50), the sublineage demonstrated efficient clonal propagation, reflecting strong hospital-based selection.

Further genomic analysis confirmed the presence of a broad resistance arsenal in this sublineage, including genes encoding multiple efflux pumps (e.g., *adeABC*, *adeFGH*) and other acquired determinants conferring resistance to aminoglycosides (e.g., *armA*), macrolides (e.g., *msrE*-*mphE*), chloramphenicol (e.g., *catB8*), disinfectants (e.g., *qacEΔ1*), and tetracyclines (e.g., *tet*(B)). Within this already resistant background, a subset of 17 isolates stood out by carrying additional acquired carbapenemase genes, such as *bla*_NDM-40_ and variant *bla*_OXA_ genes (e.g., *bla*_OXA-237_). We therefore designated these as the ‘Extended Resistome Group’ and the remaining 158 isolates, which harbored only *bla*_NDM-1_ and *bla*_OXA-23_, as the ‘Core Dual-Carbapenemase Group’ for comparative analysis.

The Extended Resistome Group showed a marginally higher mean ARG burden (65.4 vs. 63.5, Wilcoxon rank-sum test with Benjamini-Hochberg correction, adjusted *p* = 0.049) and significantly higher plasmid replicon counts (2.9 vs. 2.1, Wilcoxon rank-sum test with Benjamini-Hochberg correction, adjusted *p* < 0.001) compared to the Core Dual-Carbapenemase Group (*n* = 158) (Fig. 3B). IS counts did not differ significantly between the two groups (adjusted *p* = 0.086). Notably, in our dataset, all isolates comprising this colistin-resistant ST2 sublineage (*n* = 175) originated from the United States and were collected between 2020 and 2024. Within this temporal window, the Extended Resistome Group emerged from 2021 onward. *Ne* analysis indicated the Extended group had significantly higher effective population sizes (Wilcoxon rank-sum test, *p* = 0.01) (Fig. S3B), suggesting this subgroup is highly capable of tolerating its additional genetic load concurrent with active population expansion.

Integration of genomic and population data prompted us to hypothesize a conceptual framework regarding a "gene-load–selection" continuum (Fig. 3C). While ARGs and *Ne* showed no significant nonlinear association (quadratic regression, Adj *R*^2^ < 0.01, *p* = 0.237), plasmid replicon counts and IS elements showed significant, albeit statistically weak, nonlinear associations with *Ne* (quadratic regression, Adj *R*^2^ = 0.03, *p* = 0.034, and Adj *R*^2^ = 0.09, *p* < 0.001, respectively). The limited explanatory power of these models indicates that mobile genetic elements alone do not strictly drive population size fluctuations. Instead, this weak correlation suggests their accumulation is likely heavily intertwined with external epidemiological contexts, such as the temporal outbreaks described above.

In summary, the evolutionary trajectory of this colistin-resistant sublineage is not linear but is shaped by a dynamic interplay between gene acquisition and population dynamics. The emergence of the Extended Resistome Group, with its higher *Ne*, supports our working hypothesis that this successful lineage can not only tolerate the initial burden of an expanded resistome but can maintain its evolutionary success when navigating real-world epidemiological selections.

### Phylogenetic and ecological evidence for the clonal restriction of CRAB co-harboring *bla*_NDM-1_ and *bla*_OXA-23_

**Fig. 4:**
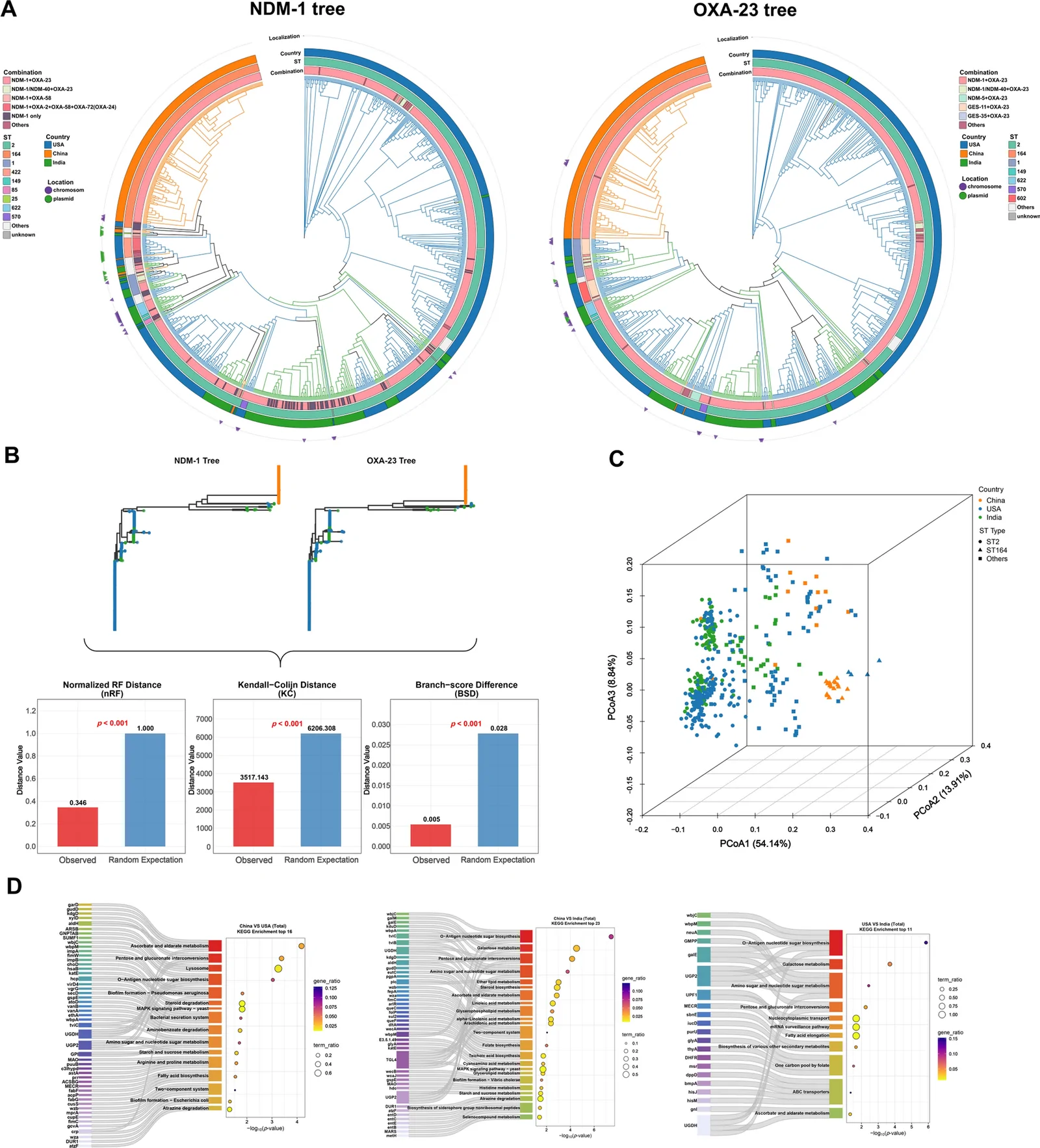
Phylogenetic concordance and geographic divergence of dual-carbapenemase CRAB clones. (In these figures, carbapenemase genotypes are represented by the corresponding enzyme names for visual clarity.) **A** Parallel core-genome phylogenies of *bla*_NDM-1_-positive (*n* = 1,032) and *bla*_OXA-23_-positive (*n* = 962) isolates from China, USA, and India. Trees were inferred from core-gene alignments and rendered as circular cladograms (branch lengths not shown). Branches are colored by country of isolation. Concentric rings (inner to outer) represent: (i) carbapenemase profile, (ii) ST, (iii) country, and (iv) location of *bla*_NDM-1_ and *bla*_OXA-23_ genes (chromosomal vs. plasmid) for genomes with complete assemblies (*n* = 46). **B** Topological congruence analysis of shared isolates (n = 909). Observed nRF (0.346), KC (3517.143), and BSD (0.005) distances between *bla*_NDM-1_-positive and *bla*_OXA-23_-positive isolate phylogenies were compared against a null distribution generated by 1000 permutations of tip labels. Continuous preservation of tree topology significantly deviated from random expectation (empirical *p* < 0.001). Rather than implying independent gene co-evolution, these metrics quantitatively confirm that the dual-carbapenemase phenotype is deeply shaped by the underlying host population structure, being rigidly restricted to specific clonal topologies rather than randomly distributed. Clone-stratified analyses for ST2 and ST164 yielded similarly structured patterns (Fig. S4B). **C** Three-dimensional PCoA based on Bray–Curtis dissimilarity of resistome-IS-plasmid profiles. PCoA1, PCoA2, and PCoA3 explain 54.1%, 13.9%, and 8.8% of variance, respectively. Each point represents an isolate, colored by country. **D** KEGG pathway enrichment analysis of differential genes from pairwise GWAS between countries. Bubble plots show pathways enriched in Chinese versus USA (left), Chinese versus India (middle), and USA versus India (right) comparisons. Bubble size represents the number of significant genes; color intensity corresponds to adjusted *p*-value. Pathway labels are provided for significant enrichments (adjusted *p* < 0.05)

To define the foundational population structure accommodating the predominant *bla*_NDM-1_ and *bla*_OXA-23_ combination, we conducted a focused core-genome phylogenetic analysis of CRAB isolates from the three most represented countries—China, the United States, and India. We reconstructed two independent ML phylogenies: one comprising 1,032 *bla*_NDM-1_-positive isolates and another comprising 962 *bla*_OXA-23_-positive isolates (Fig. 4A). The circular phylogenetic representations revealed profound lineage-specific clustering patterns. Chinese isolates formed a tightly clustered, nearly monophyletic group strictly dominated by ST164 (209/217). This pattern, consistent with minimal genetic divergence (average 6–251 SNPs), highlights a rigid and recent clonal expansion from a single source. In contrast, substantial geographic intermingling was observed between American and Indian isolates, but they were strongly confined within a broad ST2-dominated backbone. The relatively low core-genome SNP divergence between these intercontinental ST2 populations (range: 1–2812, mean: 1039.59 SNPs) further underscores ST2 as a highly conserved, dominant structural vessel.

Having established ST2 and ST164 as the dominant host clonal frameworks, we next evaluated how the two target carbapenemase genes distributed across this topology. Critically, topological congruence analysis between the phylogenies of *bla*_NDM-1_-positive and *bla*_OXA-23_-positive isolates against a null model (1000 permutations) demonstrated significant concordance (nRF = 0.346, KC = 3517.143, BSD = 0.005; all empirical *p* < 0.001; Fig. 4B). This structural consistency remained robust in clone-stratified analyses for both ST2 and ST164 (Fig. S4B). Rather than implying active gene co-evolution, this significant deviation from the null model provides topological evidence for a non-random, strict confinement of dual-carbapenemase carriage within highly specific host evolutionary trajectories. To mathematically quantify this profound clonal restriction independent of phylogenetic topology, we evaluated the distribution of resistance profiles using ecological diversity metrics. As expected, individual mobile elements (e.g., IS*Aba125*) dispersed broadly across 74 STs, yielding high diversity scores (Simpson 1-*D* = 0.768; Pielou *J* = 0.562). Strikingly, isolates exclusively harboring *bla*_NDM-1_ exhibited the highest cross-lineage mobility, disseminating across 56 distinct STs (Shannon *H* = 3.035, 1-*D* = 0.916, *J* = 0.754), while the *bla*_GES-11_ + *bla*_OXA-23_ dual-carriage control also maintained relatively even distributions (*J* = 0.705). In profound contrast, the acquisition of the *bla*_NDM-1_ + *bla*_OXA-23_ dual-phenotype triggered a mathematically stark collapse in lineage diversity. Despite constituting the largest subpopulation (*n* = 1151), this specific dual-carbapenemase profile registered the lowest diversity and evenness scores across all metrics (1-*D* = 0.614; *J* = 0.449) (Fig. S5, Table S3). Together, these topological and ecological analyses demonstrate that while individual carbapenemases like *bla*_NDM-1_ are highly promiscuous, the definitive *bla*_NDM-1_ + *bla*_OXA-23_ cassette does not diffuse randomly via unrestricted horizontal transfer; instead, it is structurally constrained and rigorously "locked" within specific accommodating clonal frameworks (primarily ST2 and ST164).

Building upon this ecological evidence of structural constraint, we further quantified the absolute penetrance of dual-carbapenemase carriage within its primary host lineages through rigorous statistical frameworks. Within the overall studied *bla*_NDM-1_-positive cohort, the ST2 and ST164 genetic backgrounds exhibited remarkably high co-occurrence rates of 91.3% and 97.7%, respectively, compared to just 52.1% across all other STs. This extraordinary enrichment is supported by profound Bayesian evidence (Bayes Factors of 7.8 × 10^19^ and 1.5 × 10^22^) and robust odds ratios (OR_ST2_ = 9.6, 95% CI: 6.0–15.4; OR_ST164_ = 38.0, 14.5–126.9). Parallel deterministic patterns with decisive Bayesian support (BF ≫ 100) were consistently confirmed across the paired *bla*_OXA-23_-positive cohort (Tables 1 and S5). Finally, hypergeometric null distribution analysis (10,000 samplings) validated that these extreme intra-lineage associations were completely distinct from stochastic sampling (empirical *p* < 0.0001; Table S4), mathematically confirming that the persistent pairing of these two genes is fiercely maintained by clone-specific forces rather than random acquisition.

**Table 1:** Statistical comparisons of carbapenemase co-occurrence patterns among ST groups

Comparison	Co-occurrence rate (%)	Odds ratio (95% CI)	Fisher's *p*-value	Bayesian factor	Evidence
A. Phylogeny of *bla*_NDM-1_-positive isolates (*n* = 1032)					
ST2 versus others	91.3 versus 52.1	9.6 (6.00–15.4)	< 0.001	7.8 × 10^19^	Overwhelming
ST164 versus others	97.7 versus 52.1	38.0 (14.5–126.9)	< 0.001	1.5 × 10^22^	Overwhelming
ST2 versus ST164	91.3 versus 97.7	0.25 (0.08–0.63)	0.001	16	Strong
B. Phylogeny of *bla*_OXA-23_-positive isolates (*n* = 962)					
ST2 versus others	97.3 versus 63.1	20.4 (10.6–40.6)	< 0.001	1.2 × 10^20^	Overwhelming
ST164 versus others	99.8 versus 63.1	∞ (29.9–∞)	< 0.001	1.9 × 10^17^	Overwhelming
ST2 versus ST164	97.3 versus 99.8	0.09 (0.02–0.70)	0.010	0.21	Inconclusive

∞ indicates non-calculable OR due to perfect co-occurrence in ST164 group; Bayesian Factor interpretation: > 100 = overwhelming, 10–30 = strong, < 1 = inconclusive

To further characterize these restricted clone backbones, accessory genome profiling illuminated the distinct evolutionary trajectories of these successful clones. ST164 possessed a unique, highly specialized resistome and mobilome, dominated by *bla*_OXA-91_, *bla*_CARB-16_, *bla*_ADC-52_, a full suite of IS*Aba* elements, and R1-type plasmids (all > 95% prevalence, log₂OR > 11). In contrast, ST2 exhibited a broad-spectrum multidrug-resistance profile featuring aminoglycoside modifiers (multiple *aph* variants), *bla*_OXA-66_, tetracycline/macrolide efflux genes, and R3-type plasmids (Fig. S6). The heterogeneous "Others" group demonstrated complementary patterns, uniquely enriched for carbapenemase genes absent in the dominant clones (*bla*_OXA-58_, *bla*_OXA-69_, *bla*_GES-11_) and carried a set of low-frequency plasmids undetected in ST2/ST164, indicating independent, less successful mobilization events outside these specific host frameworks.

To further dissect the genetic basis of *bla*_NDM-1_ acquisition in these dominant clones, we performed high-resolution genetic context analysis of completed genomes (*n* = 46), which revealed conserved but clone-specific transposon architectures (Fig. S7). In ST2 and most other STs, *bla*_NDM-1_ resided within a Tn*125*-like transposon flanked by IS*Aba125*, preserving the full groES-groEL chaperone module (*bla*_NDM-1_-*ble*_MBL_-*iso*-*tat*-*cutA*-*groES*-*groEL*). Conversely, all ST164 isolates harbored *bla*_NDM-1_ within a Tn*6924*-like backbone flanked by IS*Aba14*, lacking *groES*-*groEL*. These lineage-specific transposon architectures provide a mechanistic explanation for the independent evolutionary trajectories of ST2 and ST164, as inferred from population structure and accessory genome analysis. The convergence of both clones on the stable lineage-specific maintenance of *bla*_NDM-1_ (explored below) suggests a common adaptive solution despite their distinct genetic backgrounds.

Genomic Localization in the Complete Genome Subset. Investigation of carbapenemase location in the subset of complete genomes (*n* = 46) showed a strong clonal bias (Table S6). All available ST2 (*n* = 6) and ST164 (*n* = 3) complete isolates harbored both *bla*_NDM-1_ and *bla*_OXA-23_ on the chromosome. In contrast, minor STs, including ST422 (*n* = 7), ST126 (*n* = 2), and ST10 (*n* = 2), predominantly carried *bla*_NDM-1_ on non-mobilizable plasmids. Relaxase genes (*mobA*, *mobF*, *mobQ*) were detected on plasmids from ST2 (3/6 isolates), ST164 (2/3), and ST622 (9/10 Indian isolates co-harboring *mobA*/*mobL* and *mobF* with T4SS on conjugative plasmids). While the limited sample size (representing less than 3% of our total cohort) precludes definitive mechanistic conclusions, this pattern raises the hypothesis that the widespread dissemination of ST2 and ST164 clones may be linked to a propensity for stable vertical inheritance of key resistance determinants (potentially including chromosomal integration, as observed in this small subset), whereas sporadic lineages more frequently rely on transient plasmid acquisition—a hypothesis warranting dedicated large-scale long-read sequencing investigation.

Assessment of Prophage-Mediated Gene Transfer. To explicitly test whether the dissemination of this dual-carbapenemase genotype is driven by non-plasmid mobile genetic elements, we screened the entire cohort (*n* = 1820) for prophage-associated *bla*_NDM-1_ and *bla*_OXA-23_. Congruent with our clonal expansion model, prophage-mediated carriage was exceptionally rare, identified in only 19 isolates (~ 1.0%). Specifically, *bla*_NDM-1_ was detected within prophages in 7 isolates (belonging to minor lineages such as ST25 and ST23), while prophage-associated *bla*_OXA-23_ was found in 12 isolates (three ST164, nine ST1). Crucially, the dual-carbapenemase combination (*bla*_NDM-1_ and *bla*_OXA-23_) was never observed co-localized within a single prophage element. Notably, the globally dominant ST2 lineage exhibited zero prophage-associated carriage for either gene (Table S1). These whole-cohort findings indicate that while prophages occasionally capture individual carbapenemase genes in sporadic lineages, they are not the primary drivers of the co-dissemination of *bla*_NDM-1_ and *bla*_OXA-23_ in the dominant ST2 and ST164 clones.

Collectively, these data establish that co-carriage of *bla*_NDM-1_ and *bla*_OXA-23_ in CRAB is primarily restricted to two successful clones, ST2 and ST164, which have undergone distinct geographic expansions. Each clone maintains a coherent genetic backbone and unique accessory-genome profile, indicating that dual-enzyme resistance spreads through lineage-specific pathways. This clonal architecture provides the framework for investigating country-specific genetic refinements.

### Geographic divergence and accessory-genome stratification of dominant clones

The geographic segregation of ST164 (China) and ST2 (USA/India) as dominant dual-carbapenemase carriers (Fig. S4A) prompted us to investigate country-specific accessory-genome signatures that may underpin their regional prevalence. PCoA based on the resistome-IS-plasmid repertoire revealed clear stratification by country, with Chinese isolates forming a distinct cluster separate from the intermingled US and Indian populations (Fig. 4C). PERMANOVA confirmed that geographic origin alone explained 46.5% of the accessory-genome variance (*F* = 478.28, *p* < 0.001). When ST was included as a covariate, the model's explanatory power increased to 63.6%, with ST background independently contributing 15.4% and the country-ST interaction adding a further 1.8% (Table 2). This hierarchical nested effect—where geography dominates but is modulated by clonal background—validates our strategy to dissect country-level genetic variation while controlling for phylogenetic identity.

**Table 2:** PERMANOVA results for accessory genome variation

Model specification	Factors tested	Total *R*^2^	*F* value	*p*-value	Key finding
Model 1	Country	0.465	478.28	< 0.001	Strong country effect
Model 2	Country + ST_type	0.619	445.48	< 0.001	ST type explains additional variance
Model 3	Country × ST_type	0.636	273.72	< 0.001	Full model with interaction

PERMANOVA based on Bray–Curtis dissimilarities of accessory genome features (antibiotic resistance genes, insertion sequences, and plasmid replicons) with 9999 permutations. All tests were statistically significant (*p* < 0.001)

Unique Resistome Signatures by Country. Venn analysis of country-specific resistomes further illuminated divergent national trajectories (Fig. S8A). We quantified the diversity of distinct resistance gene types, counting each unique gene identifier (e.g., *bla*_KPC-3_, *bla*_NDM-5_) as one, irrespective of gene family. Accordingly, the USA reservoir exhibited the highest diversity (*n* = 66), including the β-lactamase genes *bla*_KPC-3_ and *bla*_NDM-5_, and *tet*(X3). In contrast, China and India possessed fewer unique genes (18 and 10, respectively), such as *bla*_NDM-2_ and *tet*(X6) in China, and *bla*_PER-1_ in India, highlighting localized acquisition patterns.

Genome-Wide Association Reveals Regional Metabolic Signatures. Pairwise GWAS between countries identified significant enrichment in ascorbate and aldarate metabolism and lysosomal pathways for China versus USA (Fig. 4D), and steroid biosynthesis and galactose metabolism for China versus India. Notably, USA versus India comparisons also highlighted galactose metabolism divergence, suggesting that regional selective pressures and/or localized clonal expansions contribute to accessory-genome divergence. Manhattan plots are provided in Figure S8B. These findings suggest that the widespread dissemination of the ST2 and ST164 clones is not a monolithic process but a hierarchically structured expansion, wherein clonal dissemination is concomitantly associated with geographically restricted genetic profiles. This geographic structuring provides the genetic basis for investigating how local ecological factors—including country-specific antimicrobial practices and regional outbreak dynamics—might shape the functional trajectory of these widespread lineages.

## Discussion

**Fig. 5:**
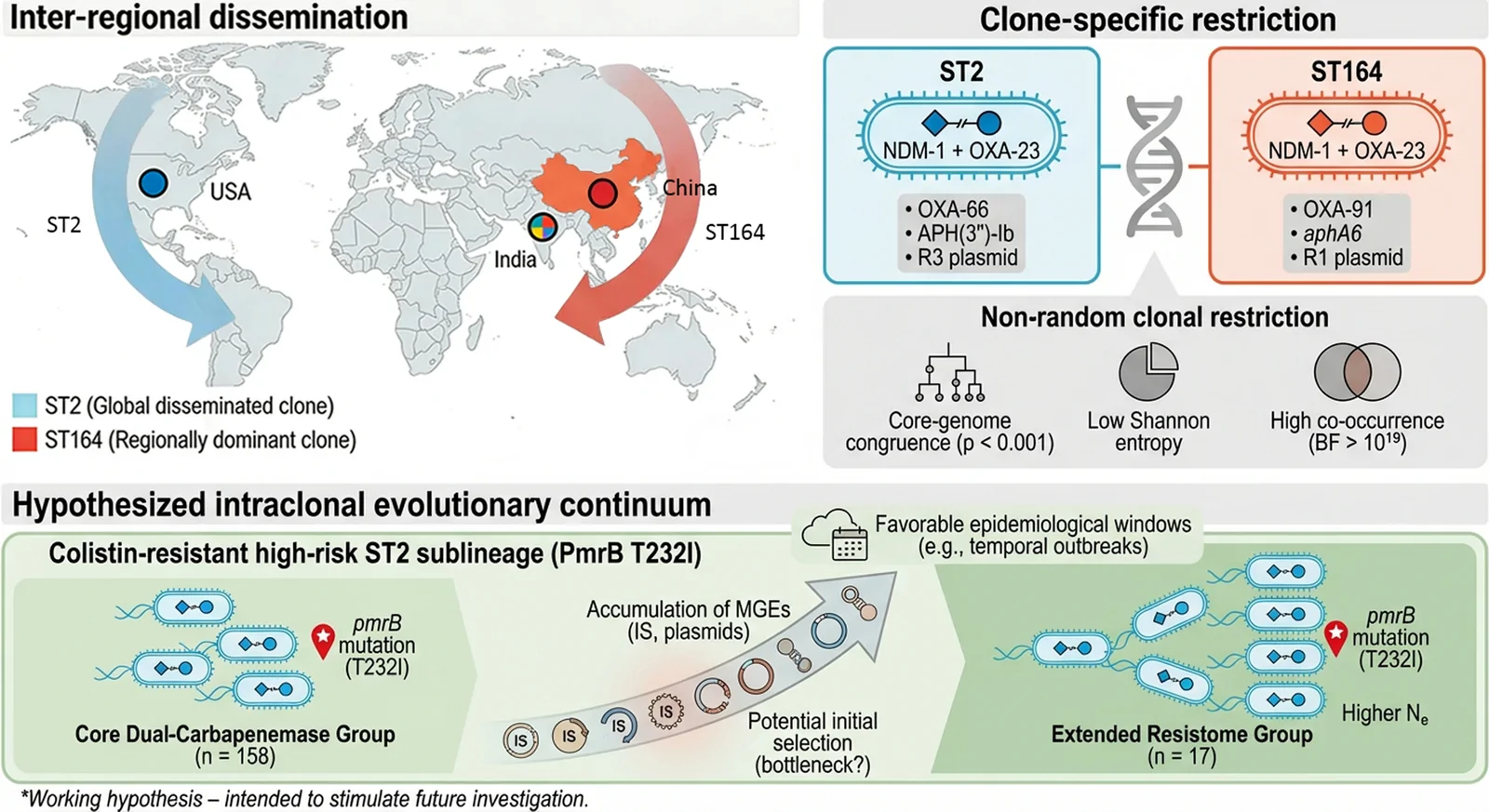
Proposed conceptual framework illustrating how clonal amplification shapes the transnational spread of CRAB associated with *bla*_NDM-1_ and *bla*_OXA-23_. (In these figures, carbapenemase genotypes are represented by the corresponding enzyme names for visual clarity.) The framework integrates three scales of analysis: inter-regional dissemination, clone-specific restriction, and intraclonal evolution. (Left) Inter-regional dissemination. The widespread dissemination is propelled by two dominant clones: widespread ST2 (ice blue) and regional ST164 (vermilion). China, USA, and India are key reservoirs. (Right) Clone-specific restriction. ST2 and ST164 convergently acquire and stabilize the *bla*_NDM-1_ and *bla*_OXA-23_ combination, despite distinct accessory genomes. A triad of evidence—high co-occurrence frequencies, low phenotypic diversity (Shannon entropy), and significant core-genome congruence (*p* < 0.001)—indicates that this dual-resistance phenotype is strongly constrained by specific clonal backbones. This highlights clonal expansion, rather than indiscriminate horizontal transfer, as the primary basis for their success at this scale. (Bottom) Hypothesized intraclonal evolutionary continuum. Within the colistin-resistant high-risk ST2 sublineage, we propose a complex interplay between genetic burden and population dynamics: gene acquisition increases load, imposing a potential initial selection (bottleneck); however, the accumulation of mobile genetic elements (MGEs) may confer the plasticity required to tolerate this burden. When aligned with favorable epidemiological conditions (e.g., temporal clinical outbreaks), clonal expansion yields an extended resistome with higher effective population size (*Ne*). This working model illustrates how permissive lineages might navigate short-term cost to achieve long-term success

The global burden of CRAB is a defining crisis in modern antimicrobial resistance. While the acquisition of mobile resistance genes is a crucial initial step, the evolutionary forces that govern the stable maintenance and widespread dissemination of high-cost, multi-carbapenemase strains remain inadequately understood. Our large-scale multinational genomic survey of 1820 isolates unveils a paradigm where the alarming success of the *bla*_NDM-1_ and *bla*_OXA-23_ dual-carbapenemase gene profile is not a story of promiscuous plasmid exchange [61], but a clonally orchestrated epidemic spread. Our data demonstrate that the extensive dissemination of the *bla*_NDM-1_ and *bla*_OXA-23_ combination is largely attributable to its stable association with specific, successful STs—primarily the internationally disseminated ST2 and the regionally-restricted ST164. Within our comprehensively characterized cohort, these dominant lineages served as the primary vehicles for this dual-carbapenemase gene profile. This evidence reinforces the critical importance of understanding resistance spread through the lens of successful global clones, rather than relying solely on gene-centric horizontal transfer models. While *A. baumannii* is increasingly recognized as a profound "One Health" threat capable of colonizing animal and diverse environmental reservoirs [62], the overwhelming clinical origin of our dual-carbapenemase cohort suggests that the widespread dissemination of the *bla*_NDM-1_/*bla*_OXA-23_ genotype is currently driven primarily by healthcare-associated transmissions. Nonetheless, continuous surveillance across human, animal, and environmental sectors remains critical to fully capture the dissemination dynamics of these highly successful clones. A conceptual framework integrating these interconnected dynamics is presented in Fig. 5.

Our first pivotal finding is that the analyzed cohort population of CRAB co-harboring *bla*_NDM-1_ and *bla*_OXA-23_ is overwhelmingly dominated by two genetically distinct clones. While the broadly existing *A. baumannii* resistome is widely recognized as highly dynamic, open, and heavily shaped by frequent promiscuous horizontal gene transfer [20, 21], the profound ecological restriction of these specific genes within specific core-genome backbones provides decisive evidence that their stable co-occurrence is intimately tied to these host frameworks. This pattern is fundamentally inconsistent with frequent horizontal transfer across diverse genetic backgrounds [63] or repeated, stochastic acquisition events characterizing the general pan-resistome. To explicitly test this, we evaluated the hypothesis that non-plasmid elements, specifically prophages, might mediate the ongoing horizontal transfer of this dual-carbapenemase genotype [44, 45]. However, comprehensive screening of the 1,820-genome cohort revealed that prophage-associated *bla*_NDM-1_ or *bla*_OXA-23_ is exceedingly rare (~ 1.0%), entirely absent in the dominant ST2 population, and the intact dual-gene combination was never identified within a single prophage. This near-absence of prophage-mediated carriage rigorously reinforces the concept that broad dissemination is not driven by continuous massive lateral transfer. Instead, this exceptionally strong clonal association, as evidenced by statistical frameworks that assign extreme statistical support (e.g., Bayes factors of 7.8 × 10^19^–1.5 × 10^22^) to the enrichment of this combination within ST2 and ST164, favors a model of clone-specific epidemiological hitchhiking. In this model, the dual-carbapenemase profile was acquired and stably maintained in ancestral ST2 and ST164 lineages, and its widespread geographic spread has been mediated primarily by the subsequent expansion of these successful clones. This model is further substantiated by the independent genetic specializations of each clone—each equipped with a unique accessory genome and distinct transposon architectures for *bla*_NDM-1_ acquisition (Tn*125*-like in ST2 vs. Tn*6924*-like in ST164) [12, 34, 64]. Thus, rather than resulting from repeated co-acquisition conferring a selective advantage, the widespread epidemiological prominence of *bla*_NDM-1_/*bla*_OXA-23_ appears to be a consequence of its integration into clonal backgrounds that themselves possess high epidemiological fitness. Finally, the chromosomal integration observed in the limited available completed genomes of these major clones, in contrast to plasmid-borne carriage in sporadic lineages, provides a preliminary glimpse into what may be a transition to stable, vertical inheritance and extensive clonal propagation. However, given that complete genomes represent only a small fraction of our dataset, further genomic surveillance is fully required to validate the universality of this chromosomal localization across the broader global population.

Delving into the epidemiologically successful ST2 clone reveals a more complex narrative of adaptation, where the relationship between genetic burden and evolutionary success is dynamically negotiated. We identified the colistin-resistant high-risk sublineage (characterized by the PmrB T232I mutation) [58, 59], which carries a significantly heavier burden of ARGs and ISs, signalling a trajectory toward pan-drug resistance. Paradoxically, within this already high-risk group, the "Extended Resistome" subgroup—bearing additional carbapenemase variants and a higher plasmid load—exhibited a larger *Ne* than the "Core" group. This counterintuitive finding challenges the canonical dogma that accessory gene acquisition universally imposes prohibitive and sustained fitness costs [65, 66]. Collectively, these intraclonal dynamics inspire a conceptual framework and working hypothesis wherein successful lineages navigate a 'gene load–selection' continuum. Within this proposed framework, we envision a cycle of gene acquisition (increasing load) → initial selection pressure (a potential bottleneck) → adaptive resolution (population recovery and differentiation) → clonal expansion (*Ne* increase). Importantly, while we observed a positive statistical association between *Ne* and the abundance of MGEs (particularly ISs), rather than with the static count of resistance genes, we recognize that the explanatory power of these nonlinear correlations remains modest (Adj *R*^2^ < 0.1). This suggests that the accumulation of mobile elements does not deterministically drive population size fluctuations on its own. Instead, the real-world expansion of these clones is likely dominated by external epidemiological contexts, such as the temporal clinical outbreaks [67] and bottleneck events described previously. Therefore, rather than acting as sheer mechanistic drivers, the profound genomic plasticity intrinsically characteristic of this pathogen likely provides the essential capacity for the 'adaptive resolution' phase [68], enabling lineages to overcome initial fitness costs and seize the opportunity to expand when favorable epidemiological windows arise. This hypothesis underscores that short-term selection pressures and long-term adaptive outcomes can be decoupled in permissive genetic backgrounds.

The transcontinental emergence of these dual-carbapenemase-carrying clonal lineages is far from a uniform process; rather, it is shaped by complex interactions between regional environmental pressures and localized transmission dynamics. The stark geographic segregation—ST164 predominantly in China versus ST2 dominating in the USA and India—provided an observational opportunity to investigate regional genomic variations. Our finding that geographic origin alone explains 46.5% of the accessory genome variance underscores the strong association between local contexts and the genetic landscape of resistance. This geographic signature is further elaborated by the GWAS-based enrichment of distinct metabolic pathways in country-specific comparisons, suggesting that distinct subpopulations may undergo divergent functional trajectories within their respective healthcare ecosystems [69–72]. However, we critically recognize that inferring regional adaptation from public genomic repositories carries inherent caveats, primarily due to geographic sampling biases [73]. As our geographic analysis was predominantly based on data from the three most represented countries (the USA, China, and India), it is susceptible to the overrepresentation of outbreak-associated isolates—a known limitation of public databases where a single successful clone spreading through a local hospital network may be sequenced disproportionately [74]. Consequently, the distinct accessory genome structures and metabolic pathway enrichments we observed via GWAS might not exclusively reflect convergent evolutionary adaptation to distinct national ecologies. Instead, these signals may also be heavily influenced by founder effects or the genetic hitchhiking of accessory traits during the rapid, localized clonal expansion of particular outbreak strains [75, 76]. While tools like Scoary utilize core-genome phylogenies to adjust for robust population structure, fully disentangling true functional adaptation from demographic outbreak dynamics remains challenging in retrospective convenience samples. Therefore, we propose that these country-specific genomic traits should currently be interpreted cautiously as valuable "regional epidemiological markers," rather than definitive proof of geographically driven adaptive fine-tuning. This hierarchical structure of the widespread dissemination—where major international clones serve as scaffolds that capture localized genomic variations—means that the threat is a mosaic of regionally-optimized sub-epidemics, necessitating surveillance strategies that are sensitive to both the clone and the context.

## Conclusion

In conclusion, our multi-dimensional analysis reveals that the transnational spread of CRAB co-harboring *bla*_NDM-1_ and *bla*_OXA-23_ is largely driven by dominant clonal lineages that have convergently adapted within high-risk healthcare settings. This repeated emergence within distinct genomic backgrounds underscores that in the evolutionary arms race, the fitness of the clone can be a more critical determinant than the potency of any single gene. Our work provides a multi-scale framework for understanding and combating such threats: broad inter-regional dissemination is channeled through a few key clones; internally, we hypothesize that these successful lineages navigate the complexities of genetic load via dynamic, potentially non-linear, evolutionary strategies; and locally, these lineages exhibit distinct geographic structuring, likely reflecting regional epidemiological dynamics. Therefore, we highlight the necessity of refining surveillance approaches—moving beyond a primary focus on resistance gene detection to also prioritize the proactive tracking and containment of these high-risk carrier clones (such as ST2 and ST164). Targeting these clones offers a more parsimonious and potentially effective strategy for dismantling the infrastructure of multidrug resistance. We acknowledge that, as with all retrospective analyses of public genome collections, our dataset is subject to geographic sampling biases and may overrepresent outbreak-associated and multidrug-resistant isolates, which could influence fine-scale estimates of prevalence and expansion dynamics. Despite these limitations, the central finding—that this widespread threat is clonally propagated by the convergent expansion of ST2 and ST164—remains strongly supported by the available data, providing a practical epidemiological framework for more targeted and effective intervention.

## Supplementary Information

Supplementary Material 1.

Supplementary Material 2.

Supplementary Material 3.

Supplementary Material 4.

## Data Availability

The datasets used and/or analysed during the current study are available from the corresponding author on reasonable request.
