# Supplementary material for: Convergent evolution of ST2 and ST164 mediates dissemination of the blaNDM-1/blaOXA-23 profile in Acinetobacter baumannii
Source: Ann Clin Microbiol Antimicrob. 2026 Jun 22;25:35. doi: 10.1186/s12941-026-00872-5 (PMC13584254; doi:10.1186/s12941-026-00872-5)
Supplement: Supplementary file 1 [file 12941_2026_872_MOESM2_ESM.pdf]

Figure S1

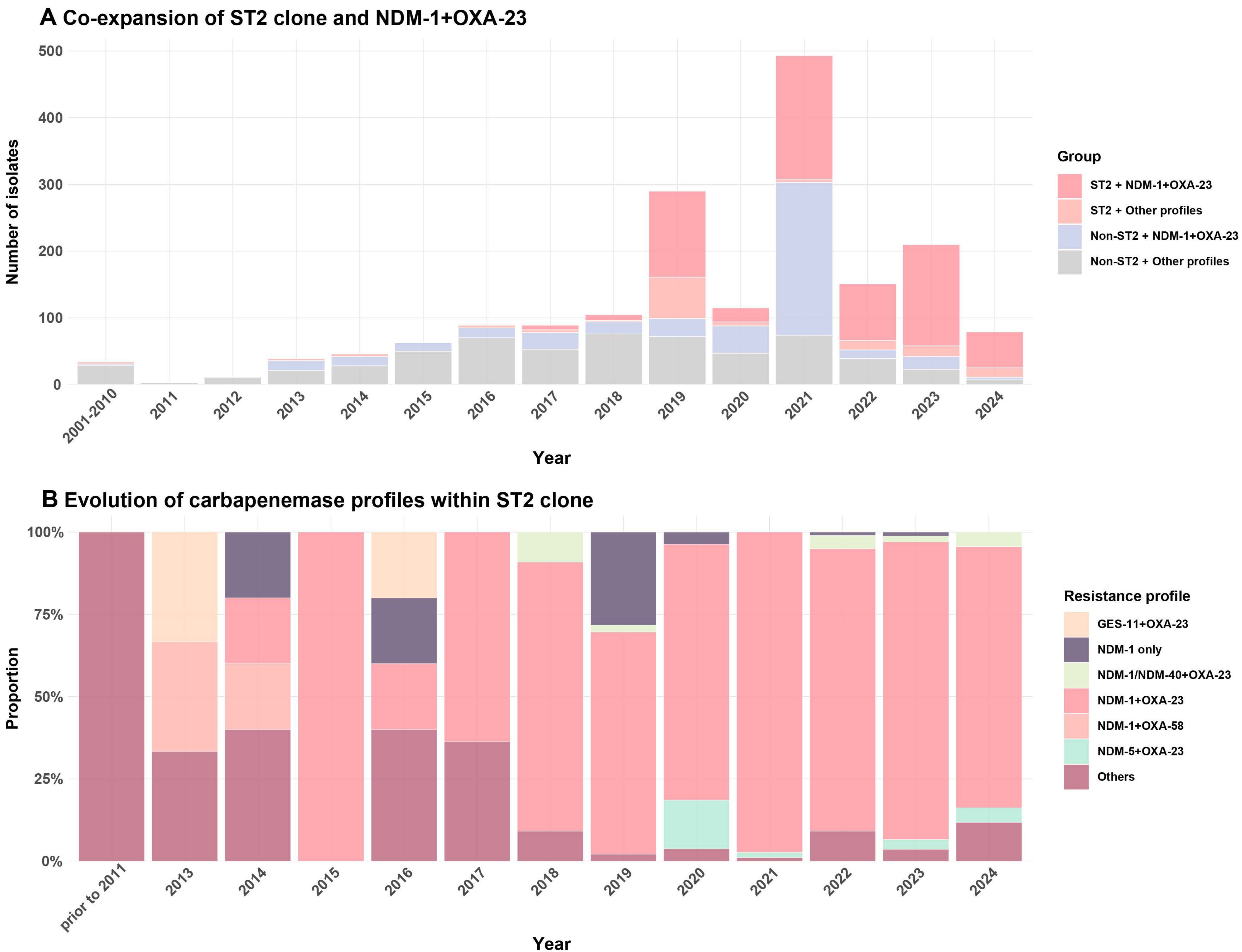

A Co-expansion of ST2 clone and NDM-1+OXA-23
Number of isolates
0
100
200
300
400
500
2001-2010
2011
2012
2013
2014
2015
2016
2017
2018
2019
2020
2021
2022
2023
2024
Year
Group
ST2 + NDM-1+OXA-23
ST2 + Other profiles
Non-ST2 + NDM-1+OXA-23
Non-ST2 + Other profiles
B Evolution of carbapenemase profiles within ST2 clone
Proportion
0%
25%
50%
75%
100%
prior to 2011
2013
2014
2015
2016
2017
2018
2019
2020
2021
2022
2023
2024
Year
Resistance profile
GES-11+OXA-23
NDM-1 only
NDM-1/NDM-40+OXA-23
NDM-1+OXA-23
NDM-1+OXA-58
NDM-5+OXA-23
Others

Figure S2

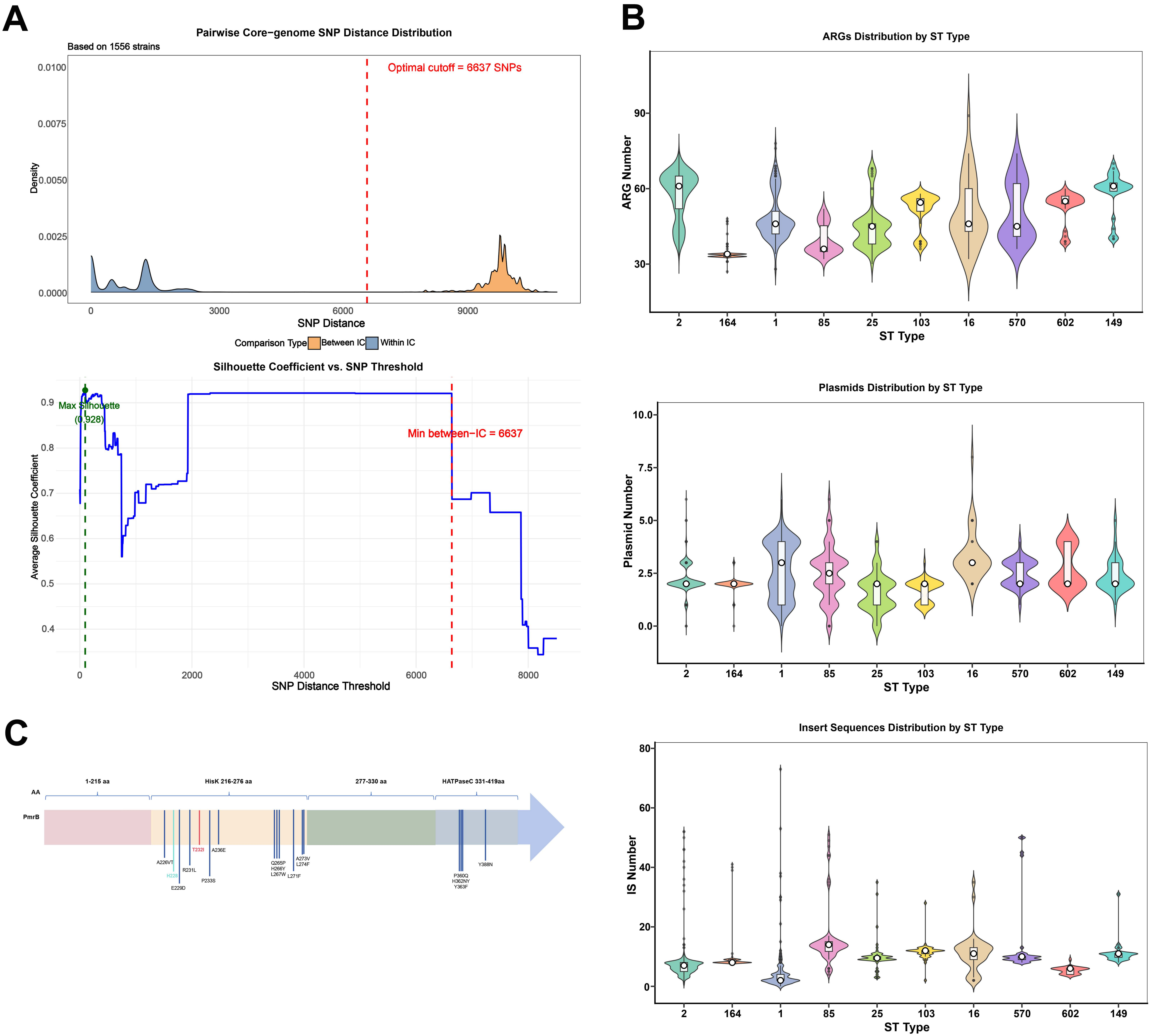

A
Pairwise Core−genome SNP Distance Distribution
Based on 1556 strains
Optimal cutoff = 6637 SNPs
Density
SNP Distance
Comparison Type
Between IC
Within IC
Silhouette Coefficient vs. SNP Threshold
Max Silhouette
(0.928)
Min between−IC = 6637
Average Silhouette Coefficient
SNP Distance Threshold
B
ARGs Distribution by ST Type
ARG Number
ST Type
Plasmids Distribution by ST Type
Plasmid Number
ST Type
Insert Sequences Distribution by ST Type
IS Number
ST Type
C
1-215 aa
HisK 216-276 aa
277-330 aa
HATPaseC 331-419aa
AA
PmrB
A226VT
H228
E229D
R231L
T232I
P233S
A236E
Q265P
H266Y
L267W
L271F
A273V
L274F
P360Q
H362NY
Y363F
Y388N

Figure S3

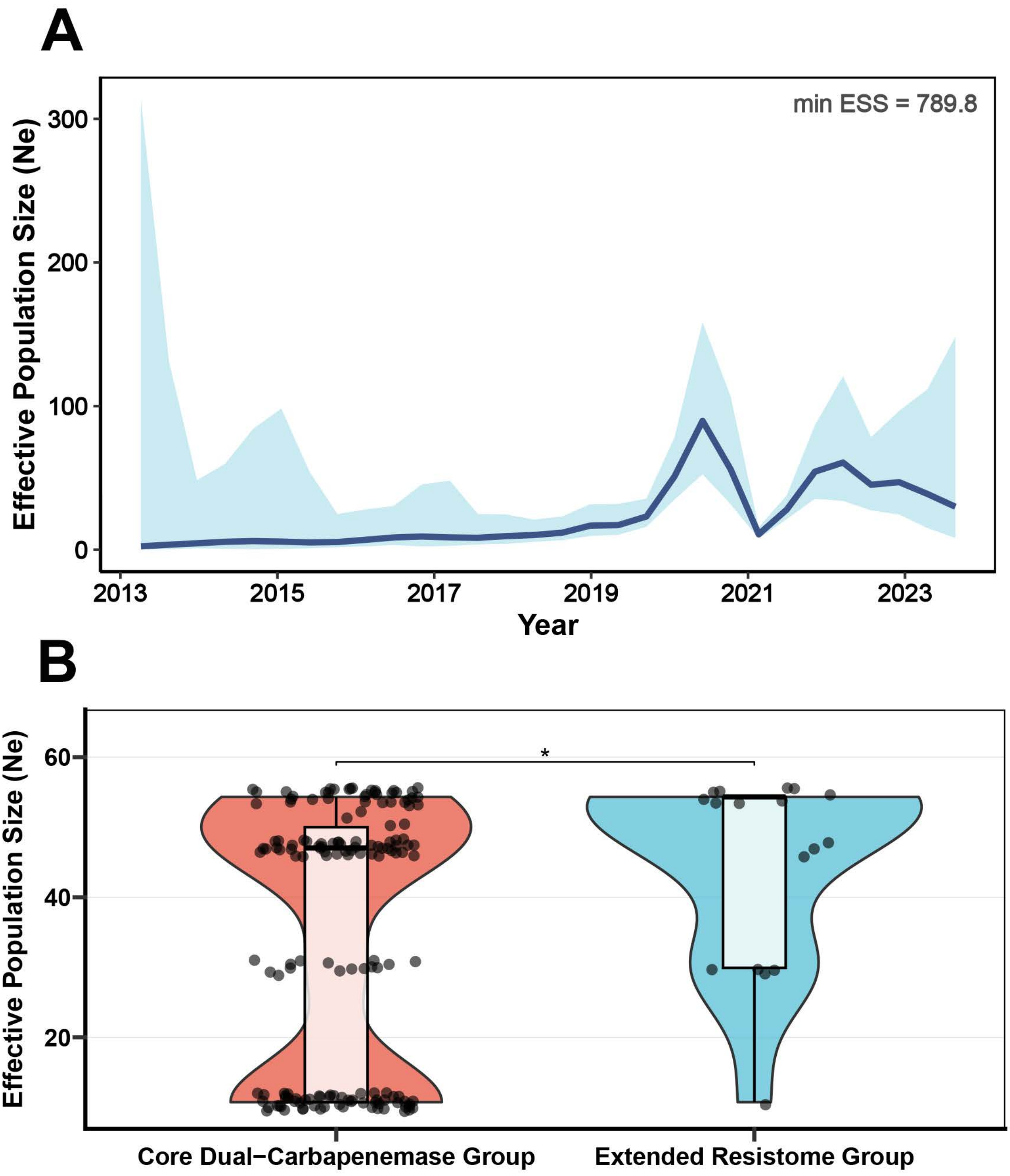
A
min ESS = 789.8
Effective Population Size (Ne)
300
200
100
0
2013
2015
2017
2019
2021
2023
Year
B
Effective Population Size (Ne)
60
40
20
*
Core Dual−Carbapenemase Group
Extended Resistome Group

Figure S4

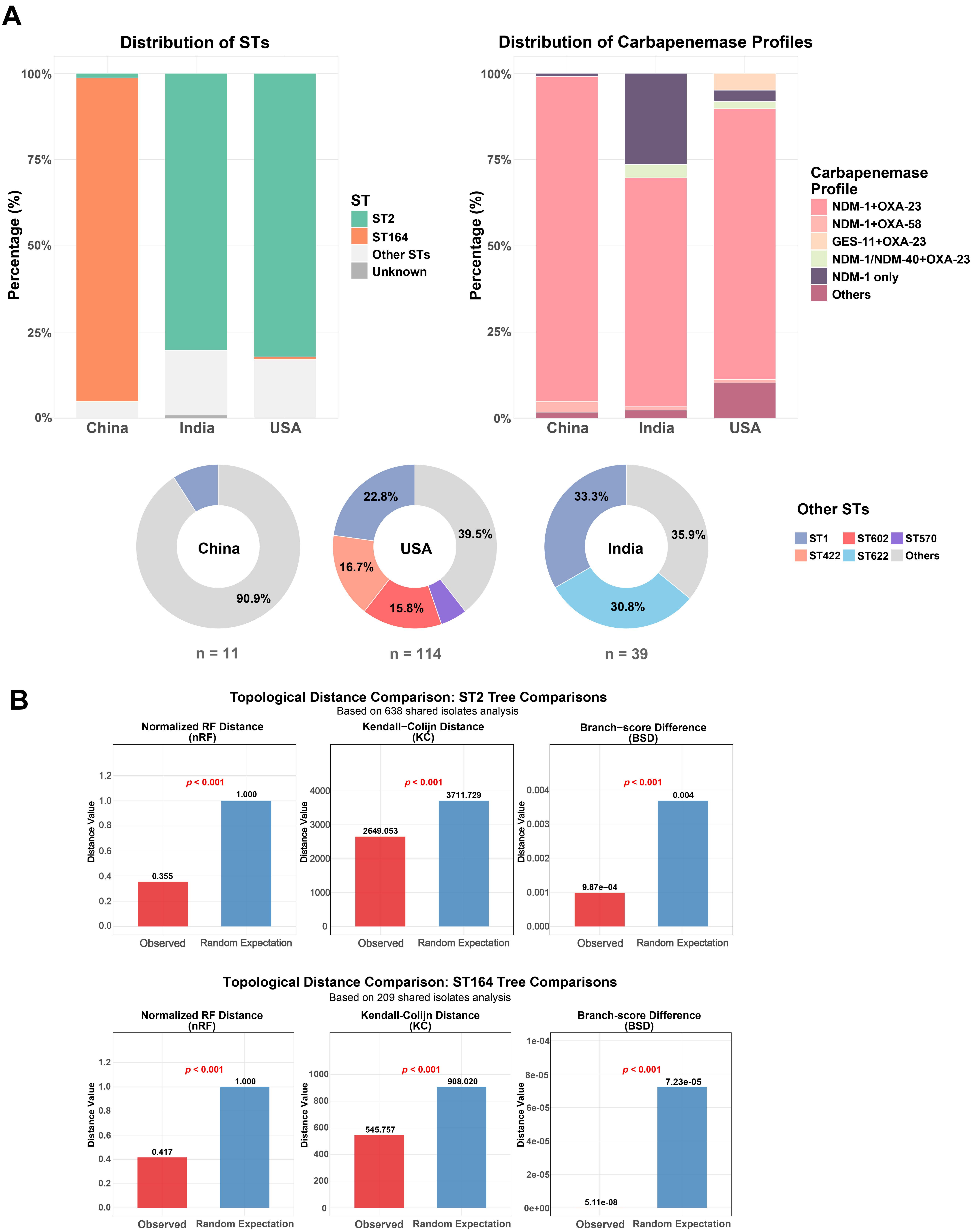
A
Distribution of STs
100%
75%
50%
25%
0%
Percentage (%)
China
India
USA
ST
ST2
ST164
Other STs
Unknown
Distribution of Carbapenemase Profiles
Carbapenemase Profile
NDM-1+OXA-23
NDM-1+OXA-58
GES-11+OXA-23
NDM-1/NDM-40+OXA-23
NDM-1 only
Others
China
90.9%
n = 11
USA
22.8%
39.5%
16.7%
15.8%
n = 114
India
33.3%
35.9%
30.8%
n = 39
Other STs
ST1
ST602
ST570
ST422
ST622
Others
B
Topological Distance Comparison: ST2 Tree Comparisons
Based on 638 shared isolates analysis
Normalized RF Distance (nRF)
Kendall−Colijn Distance (KC)
Branch−score Difference (BSD)
Distance Value
p < 0.001
0.355
1.000
2649.053
3711.729
9.87e−04
0.004
Observed
Random Expectation
Topological Distance Comparison: ST164 Tree Comparisons
Based on 209 shared isolates analysis
Normalized RF Distance (nRF)
Kendall-Colijn Distance (KC)
Branch-score Difference (BSD)
Distance Value
p < 0.001
0.417
1.000
545.757
908.020
5.11e-08
7.23e-05
Observed
Random Expectation

Figure S5

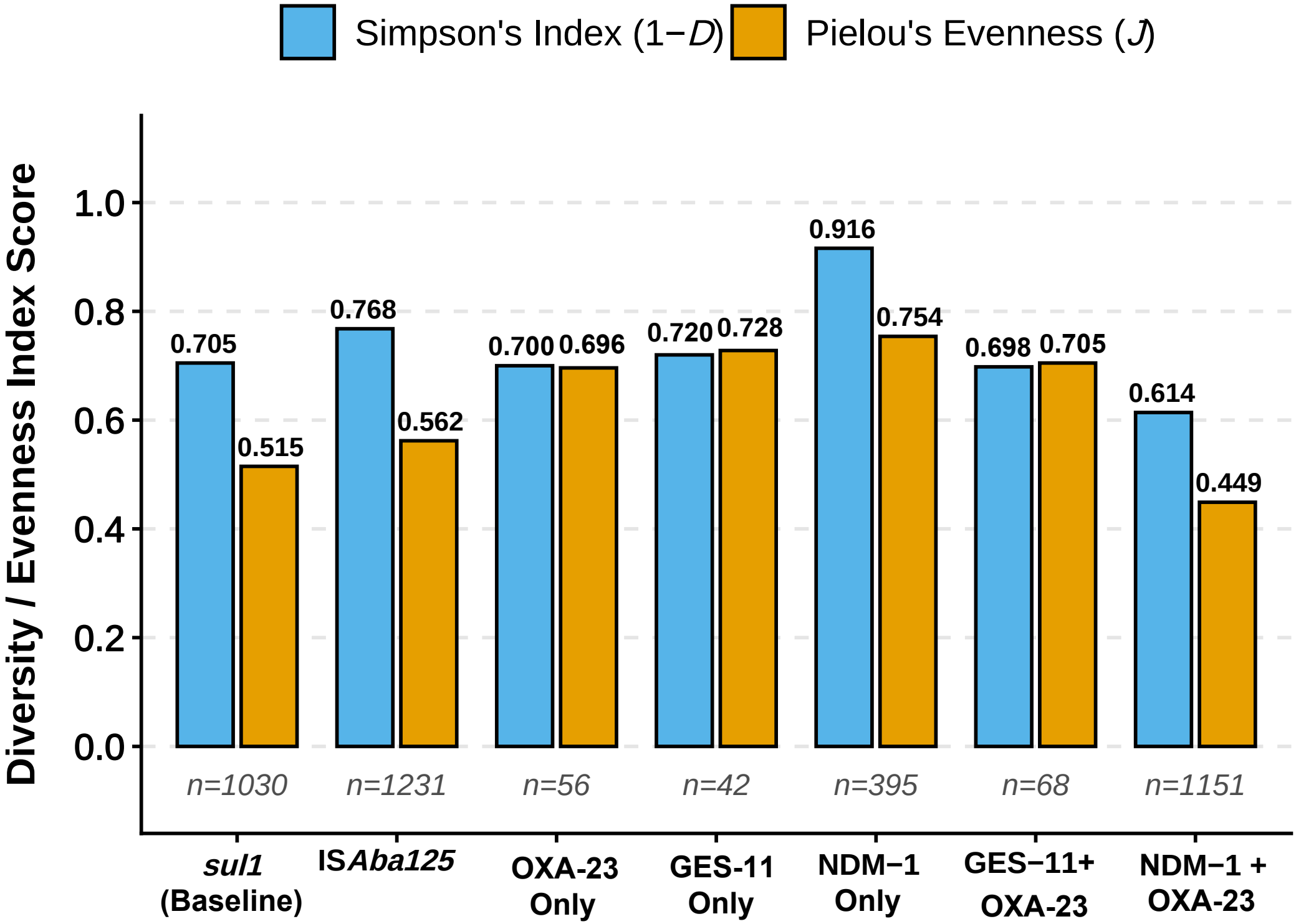

Simpson's Index (1−D)
Pielou's Evenness (J)
Diversity / Evenness Index Score
1.0
0.8
0.6
0.4
0.2
0.0
0.705
0.515
0.768
0.562
0.700
0.696
0.720
0.728
0.916
0.754
0.698
0.705
0.614
0.449
n=1030
n=1231
n=56
n=42
n=395
n=68
n=1151
sul1 (Baseline)
ISAba125
OXA-23 Only
GES-11 Only
NDM−1 Only
GES−11+ OXA-23
NDM−1 + OXA-23

Figure S6

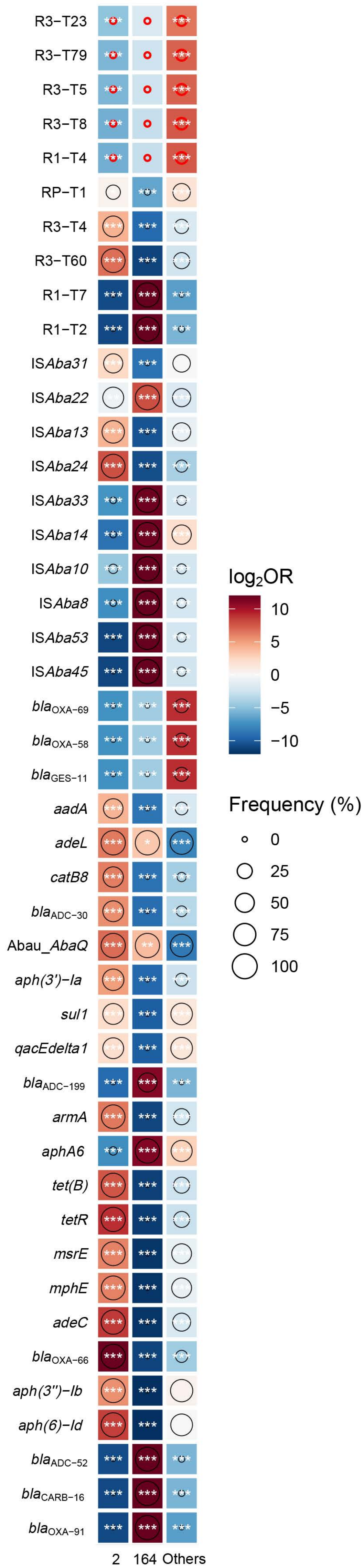
R3−T23
R3−T79
R3−T5
R3−T8
R1−T4
RP−T1
R3−T4
R3−T60
R1−T7
R1−T2
ISAba31
ISAba22
ISAba13
ISAba24
ISAba33
ISAba14
ISAba10
ISAba8
ISAba53
ISAba45
blaOXA−69
blaOXA−58
blaGES−11
aadA
adeL
catB8
blaADC−30
Abau_AbaQ
aph(3')−Ia
sul1
qacEdelta1
blaADC−199
armA
aphA6
tet(B)
tetR
msrE
mphE
adeC
blaOXA−66
aph(3'')−Ib
aph(6)−Id
blaADC−52
blaCARB−16
blaOXA−91
2
164
Others
log2OR
10
5
0
−5
−10
Frequency (%)
0
25
50
75
100

Figure S7

A

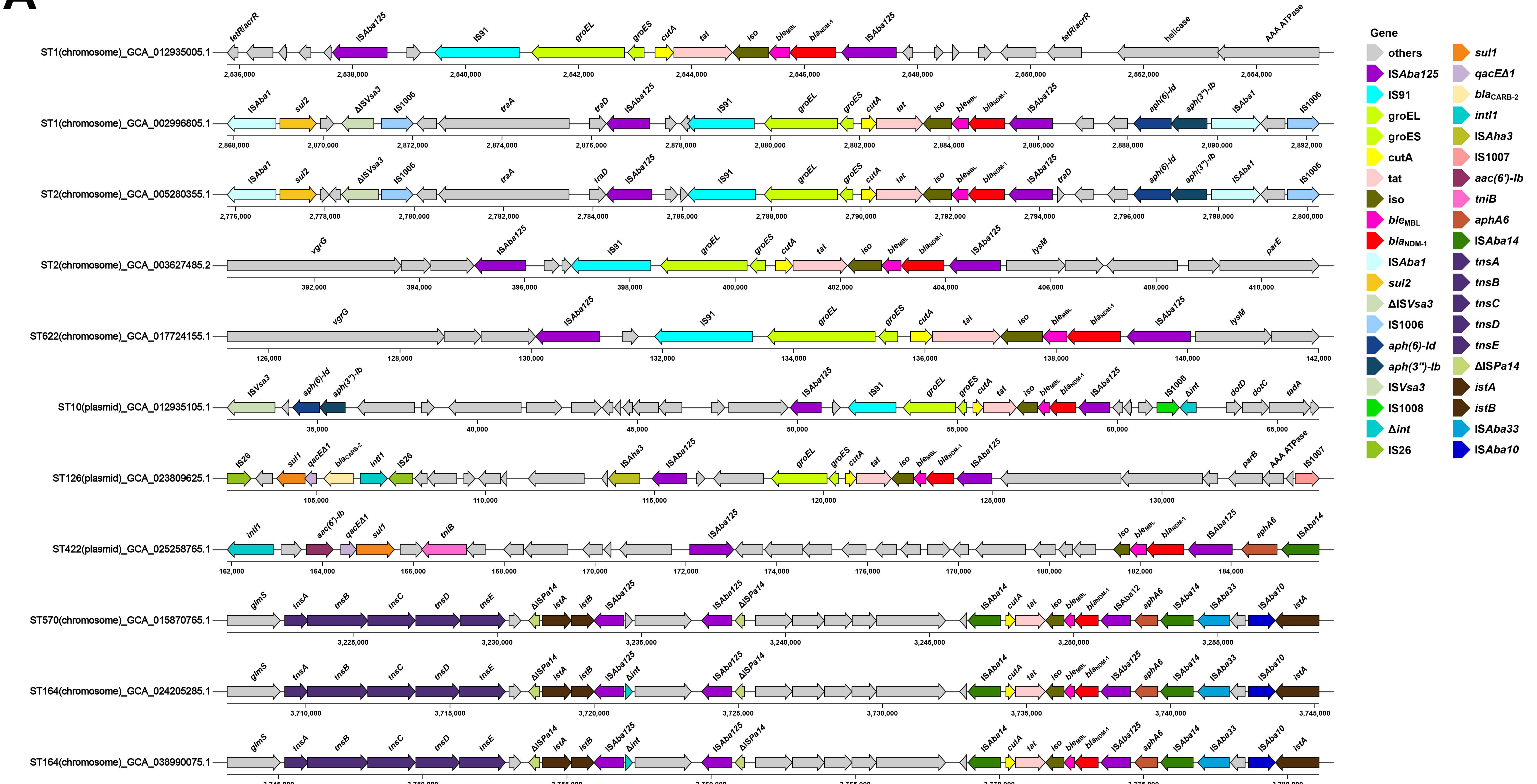

ST1(chromosome)_GCA_012935005.1
ST1(chromosome)_GCA_002996805.1
ST2(chromosome)_GCA_005280355.1
ST2(chromosome)_GCA_003627485.2
ST622(chromosome)_GCA_017724155.1
ST10(plasmid)_GCA_012935105.1
ST126(plasmid)_GCA_023809625.1
ST422(plasmid)_GCA_025258765.1
ST570(chromosome)_GCA_015870765.1
ST164(chromosome)_GCA_024205285.1
ST164(chromosome)_GCA_038990075.1
Gene
others
ISAba125
IS91
groEL
groES
cutA
tat
iso
ble_MBL
bla_NDM-1
ISAba1
sul2
ΔISVsa3
IS1006
aph(6)-Id
aph(3'')-Ib
ISVsa3
IS1008
Δint
IS26
sul1
qacEΔ1
bla_CARB-2
intI1
ISAha3
IS1007
aac(6')-Ib
tniB
aphA6
ISAba14
tnsA
tnsB
tnsC
tnsD
tnsE
ΔISPa14
istA
istB
ISAba33
ISAba10

B

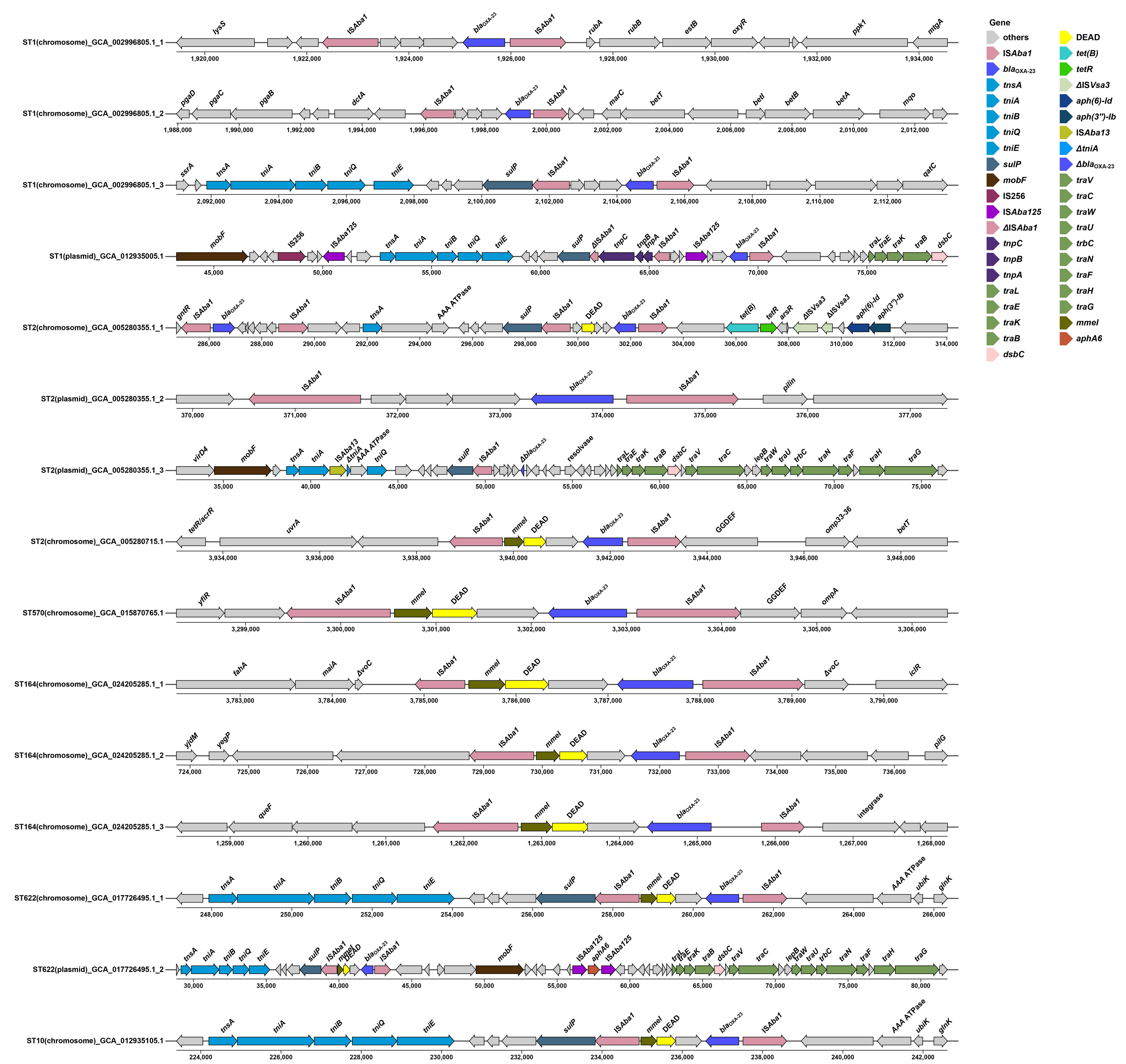

ST1(chromosome)_GCA_002996805.1_1
ST1(chromosome)_GCA_002996805.1_2
ST1(chromosome)_GCA_002996805.1_3
ST1(plasmid)_GCA_012935005.1
ST2(chromosome)_GCA_005280355.1_1
ST2(plasmid)_GCA_005280355.1_2
ST2(plasmid)_GCA_005280355.1_3
ST2(chromosome)_GCA_005280715.1
ST570(chromosome)_GCA_015870765.1
ST164(chromosome)_GCA_024205285.1_1
ST164(chromosome)_GCA_024205285.1_2
ST164(chromosome)_GCA_024205285.1_3
ST622(chromosome)_GCA_017726495.1_1
ST622(plasmid)_GCA_017726495.1_2
ST10(chromosome)_GCA_012935105.1
Gene
others
ISAba1
bla_OXA-23
tnsA
tniA
tniB
tniQ
tniE
sulP
mobF
IS256
ISAba125
ΔISAba1
tnpC
tnpB
tnpA
traL
traE
traK
traB
dsbC
DEAD
tet(B)
tetR
ΔISVsa3
aph(6)-Id
aph(3'')-Ib
ISAba13
ΔtniA
Δbla_OXA-23
traV
traC
traW
traU
trbC
traN
traF
traH
traG
mmel
aphA6

Figure S8

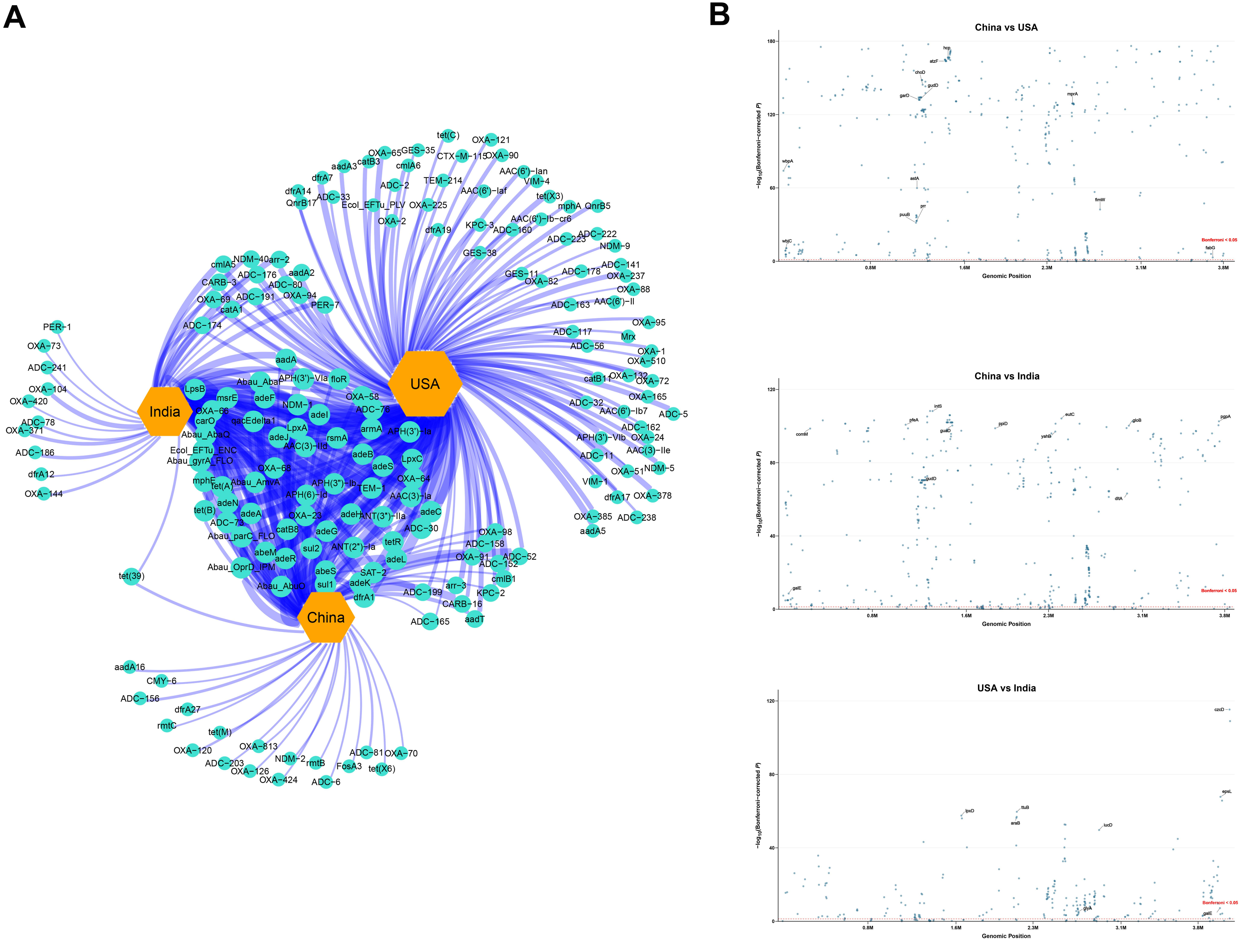
A
B
India
USA
China
China vs USA
China vs India
USA vs India
Genomic Position
Bonferroni < 0.05
