# Supplementary material for: Convergent evolution of ST2 and ST164 mediates dissemination of the blaNDM-1/blaOXA-23 profile in Acinetobacter baumannii
Source: Ann Clin Microbiol Antimicrob. 2026 Jun 22;25:35. doi: 10.1186/s12941-026-00872-5 (PMC13584254; doi:10.1186/s12941-026-00872-5)
Supplement: Supplementary file 2 [file 12941_2026_872_MOESM1_ESM.docx]

Supplementary Figure S1. Temporal dynamics of the ST2 clone and the *bla*_NDM-1_ and *bla*_OXA-23_ profile. (In these figures, carbapenemase genotypes are represented by the corresponding enzyme names for visual clarity.) (A) Population-level coordination. Stacked-bar plot comparing annual isolate counts (y-axis) over time (x-axis), spanning the initial period '2001–2010' to the year 2024. Data are categorized into four groups: ST2 isolates co-harboring *bla*_NDM-1_ and *bla*_OXA-23_, ST2 isolates with other carbapenemase profiles, non-ST2 isolates co-harboring *bla*_NDM-1_ and *bla*_OXA-23_, and non-ST2 isolates with other profiles. (B) Within-ST2 evolution of resistance combinations. Stacked-bar plot showing the percentage distribution (y-axis) of resistance profiles over time (x-axis) for the ST2 lineage only, from 'Prior to 2011' to 2024. Bars show the relative abundance of the *bla*_NDM-1_ and *bla*_OXA-23_ profile versus all other resistance combinations carried by ST2 isolates.

Supplementary Figure S2. Population-structure definition and accessory-genome features of major STs. (A) Silhouette analysis of pairwise core-genome SNP distances defining International Clone boundaries (optimal threshold 95 SNPs; max. silhouette = 0.928; minimum inter-IC distance 6,637 SNPs). (B) Accessory-genome burden of the top eight STs. Violin plots show the distribution of ARG, IS and plasmid replicon counts. ****p* < 0.001, ***p* < 0.01 (Wilcoxon rank-sum test). (C) PmrB amino-acid variation across 1,820 M-CRAb isolates. Domain annotation: HisK (histidine kinase) and HATPaseC (ATPase-like); conserved His-228 is highlighted in light blue, the high-risk T232I substitution in red.

Supplementary Figure S3. Population dynamics of the colistin-resistant high-risk ST2 sublineage. (A) Effective population size (*Ne*) trajectory estimated using the Skygrowth method for the 175-isolate pmrB-T232I sublineage. The solid line represents the median *Ne* estimate, with the shaded area indicating the 95% highest posterior density (HPD) interval. (B) Comparative effective population size between ST2 subgroups. Violin plots show the distribution of *Ne* between the "Core Dual-Carbapenemase Group" (*n* = 158) and the "Extended Resistome Group" (*n* = 17). The Extended Group exhibits a significantly higher median *Ne* (Wilcoxon rank‑sum test, *p* = 0.01).

Supplementary Figure S4. Clonal stratification of dual-carbapenemase carriers in the three-country cohort, related to Figure 4. (In these figures, carbapenemase genotypes are represented by the corresponding enzyme names for visual clarity.) (A) Population structure and resistance profiles. Stacked bar plots depict the distribution of sequence types (STs, left panel) and carbapenemase gene combinations (right panel) among CRAB isolates from China, USA, and India. Pie charts show the proportional composition of minor STs beyond the dominant clone in each country. The data quantify the near-complete overlap of the ST164 clone with the co-occurrence of *bla*_NDM-1_ and *bla*_OXA-23_ in China, and the strong association of ST2 with this dual-gene combination in the USA and India. (B) Clone-specific phylogenetic congruence. Topological congruence analysis, as in Figure 4B, performed on phylogenies pruned to include only shared isolates within the (i) ST2 (*n* = 638) and (ii) ST164 (*n* = 209) lineages. For both major clones, the observed distances (nRF, KC, BSD) for the comparison between *bla*_NDM-1_-positive and *bla*_OXA-23_-positive isolate phylogenies were significantly lower than the null expectation generated by tip-label permutation (empirical *p* < 0.001). By rejecting the null hypothesis of random distribution, these metrics demonstrate that the dual-carbapenemase phenotype remains tightly constrained by the underlying intra-clonal population structure. This structural rigidity confirms that the dual-gene cassette is non-randomly restricted to specific genetic sub-lineages within these major clones, strongly supporting the proposed model of clone-specific maintenance over frequent horizontal acquisition.

Supplementary Figure S5. Ecological diversity indices across different resistance profiles within the entire isolate cohort. (In these figures, carbapenemase genotypes are represented by the corresponding enzyme names for visual clarity.) A double bar chart illustrating Simpson’s Diversity Index (1-*D*, blue bars) and Pielou’s Evenness Index (*J*, orange bars), calculated based on the complete study dataset (*n* = 1,820 isolates). The cohort was stratified across seven distinct horizontally acquired genetic features: the baseline resistance gene (*sul1*), the common transposition vehicle (IS*Aba125*), single carbapenemase carriage (*bla*_OXA-23_, *bla*_GES-11_, or *bla*_NDM-1_), and dual carbapenemase carriage (*bla*_GES-11_ + *bla*_OXA-23_ vs. *bla*_NDM-1_ + *bla*_OXA-23_). Numerical values above the bars represent precise calculated indices, while the specific sample size (*n*) for each genetic profile is denoted below the axis. Decreased Simpson's and Pielou's scores in the *bla*_NDM-1_ + *bla*_OXA-23_ group reflect a marked reduction in overall sequence type (ST) diversity and distribution evenness, corroborating the hypothesis of lineage-associated distribution rather than random dissemination.

Supplementary Figure S6. Accessory-genome coupling of epidemic clones ST2, ST164, and other STs. The dominant ST2 and ST164 clones possess distinct, mutually exclusive arsenals of accessory genes, while the "Others" group employs alternative resistance and mobilization strategies. The heatmap visualizes the enrichment of accessory genes (ARGs, ISs, plasmid replicons) across these groups (*n* = 1,104). Features were included based on prevalence (≥20% in any ST group) or significance (*p* < 0.05 with |log₂OR| ≥ 2). Cell color indicates the log₂ odds ratio (red: enrichment; blue: depletion). Circle size corresponds to feature prevalence (%). A red outline denotes features that are both significant and unique to the "Others" group. Significance levels: **p* < 0.05, ***p* < 0.01, ****p* < 0.001 (Fisher's exact test with Benjamini–Hochberg correction).

Supplementary Figure S7. Genetic context of carbapenemase genes in representative complete genomes. (A) Flanking genetic architecture of *bla*_NDM-1_. Schematic representation of the *bla*_NDM-1_ genetic environment for representative isolates from major sequence types. The structure *bla*_NDM-1_-*ble*_MBL_-*iso*-*tat*-*cutA*-*groES*-*groEL* within a Tn*125*-like transposon, flanked by IS*Aba125*, is conserved in ST2 and most other lineages. ST164 carries *bla*_NDM-1_ within a distinct Tn*6924*-like transposon flanked by IS*Aba14*, lacking the *groES*-*groEL* module. (B) Flanking genetic architecture of *bla*_OXA-23_. Schematic representation of the *bla*_OXA-23_ genetic context in representative isolates. Arrows indicate the direction of transcription. Analysis of these structures reveals lineage-specific mobilization platforms for both carbapenemase genes.

Supplementary Figure S8. Country-specific resistome repertoires and GWAS Manhattan plots. (A) Venn diagram of antibiotic resistance gene (ARG) distribution. Diagram illustrating the shared and unique resistomes of CRAB isolates from China, USA, and India. The United States reservoir harbors the highest number of unique ARGs (*n* = 66), while China and India possess 18 and 10 unique genes, respectively. A core set of 60 ARGs is shared among all three countries. (B) Manhattan plots for pairwise country comparisons (top: China vs. USA; middle: China vs. India; bottom: USA vs. India). Each point represents a gene; its vertical position shows -log₁₀(Bonferroni-corrected *p* value). The horizontal dashed line marks the genome-wide significance threshold (Bonferroni-corrected *p* < 0.05). Genes above this line are significantly enriched in the indicated country comparison.
