# Supplementary material for: Convergent evolution of ST2 and ST164 mediates dissemination of the blaNDM-1/blaOXA-23 profile in Acinetobacter baumannii
Source: Ann Clin Microbiol Antimicrob. 2026 Jun 22;25:35. doi: 10.1186/s12941-026-00872-5 (PMC13584254; doi:10.1186/s12941-026-00872-5)
Supplement: Supplementary file 3 [file 12941_2026_872_MOESM3_ESM.docx]

Table S3. Comprehensive statistical validation

A. Overall association tests

| Dataset^a^ | Test | Statistic | df | *p*-value |
| --- | --- | --- | --- | --- |
| *bla*_NDM-1_+ phylogeny (n = 1,032) | Pearson's Chi-squared | 172.3 | 2 | < 2.2×10^-16^ |
| *bla*_OXA-23_+ phylogeny (n = 962) | Pearson's Chi-squared | 203.6 | 2 | < 2.2×10^-16^ |

Footnotes: ^a^*bla*_NDM-1_+ phylogeny and *bla*_OXA-23_+ phylogeny denote the maximum-likelihood phylogenies reconstructed from the core genomes of isolates positive for the *bla*_NDM-1_ and *bla*_OXA-23_ genes, respectively.

B. Null distribution analysis (10,000 hypergeometric samplings)

| ST Group | Dataset^a^ | Empirical *p*-value | Interpretation |
| --- | --- | --- | --- |
| ST2 | *bla*_NDM-1_+ phylogeny | < 0.0001 | Non-random pattern |
| ST164 | *bla*_NDM-1_+ phylogeny | < 0.0001 | Non-random pattern |
| ST2 | *bla*_OXA-23_+ phylogeny | < 0.0001 | Non-random pattern |
| ST164 | *bla*_OXA-23_+ phylogeny | < 0.0001 | Non-random pattern |

Footnotes: ^a^*bla*_NDM-1_+ phylogeny and *bla*_OXA-23_+ phylogeny denote the maximum-likelihood phylogenies reconstructed from the core genomes of isolates positive for the *bla*_NDM-1_ and *bla*_OXA-23_ genes, respectively.

Table S4. Sample characteristics and co-occurrence metrics by ST group

| ST Group^a^ | Total Isolates | Co-occurring Isolates | Co-occurrence Rate (%) | 95% CI | Bayes Factor vs Others |
| --- | --- | --- | --- | --- | --- |
| *bla*_NDM-1_+ phylogeny **(n = 1,032)** |  | | | | |
| ST2 | 699 | 638 | 91.3 | 89.1 - 93.5 | 7.8×10^19^ |
| ST164 | 214 | 209 | 97.7 | 95.2 - 99.8 | 1.5×10^22^ |
| Others | 119 | 62 | 52.1 | 43.2 - 61.0 | 1 |
| *bla*_OXA-23_+ phylogeny **(n = 962)** |  | | | | |
| ST2 | 655 | 638 | 97.3 | 96.0 - 98.6 | 1.2×10^20^ |
| ST164 | 209 | 209 | 99.8 | 99.5 - 100.0 | 1.9×10^17^ |
| Others | 98 | 62 | 63.3 | 53.7 - 72.9 | 1 |

Footnotes: ^a^*bla*_NDM-1_+ phylogeny and *bla*_OXA-23_+ phylogeny denote the maximum-likelihood phylogenies reconstructed from the core genomes of isolates positive for the *bla*_NDM-1_ and *bla*_OXA-23_ genes, respectively.
